# Evolutionary Quantitative Proteomics of Reproductive Protein Divergence in *Drosophila*

**DOI:** 10.1016/j.mcpro.2023.100610

**Published:** 2023-06-28

**Authors:** Martin D. Garlovsky, Yasir H. Ahmed-Braimah

**Affiliations:** Department of Biology, Syracuse University, Syracuse, New York, USA

**Keywords:** seminal fluid proteins, female reproductive tract, *Drosophila*, quantitative proteomics, speciation, postmating prezygotic isolation

## Abstract

Reproductive traits often evolve rapidly between species. Understanding the causes and consequences of this rapid divergence requires characterization of female and male reproductive proteins and their effect on fertilization success. Species in the *Drosophila virilis* clade exhibit rampant interspecific reproductive incompatibilities, making them ideal for studies on diversification of reproductive proteins and their role in speciation. Importantly, the role of intraejaculate protein abundance and allocation in interspecific divergence is poorly understood. Here, we identify and quantify the transferred male ejaculate proteome using multiplexed isobaric labeling of the lower female reproductive tract before and immediately after mating using three species of the *virilis* group. We identified over 200 putative male ejaculate proteins, many of which show differential abundance between species, suggesting that males transfer a species-specific allocation of seminal fluid proteins during copulation. We also identified over 2000 female reproductive proteins, which contain female-specific serine-type endopeptidases that showed differential abundance between species and elevated rates of molecular evolution, similar to that of some male seminal fluid proteins. Our findings suggest that reproductive protein divergence can also manifest in terms of species-specific protein abundance patterns.

## Highlights

• We simultaneously characterize the male and female reproductive proteome.
• We identified >200 ejaculate proteins and >2000 female reproductive tract proteins.
• Many proteins show species-specific abundances.
• Differentially abundant proteins include many belonging to multicopy gene families.
• Female serine-type endopeptidases show differential abundance and evolve quickly.

## Graphical Abstract

**Figure:**
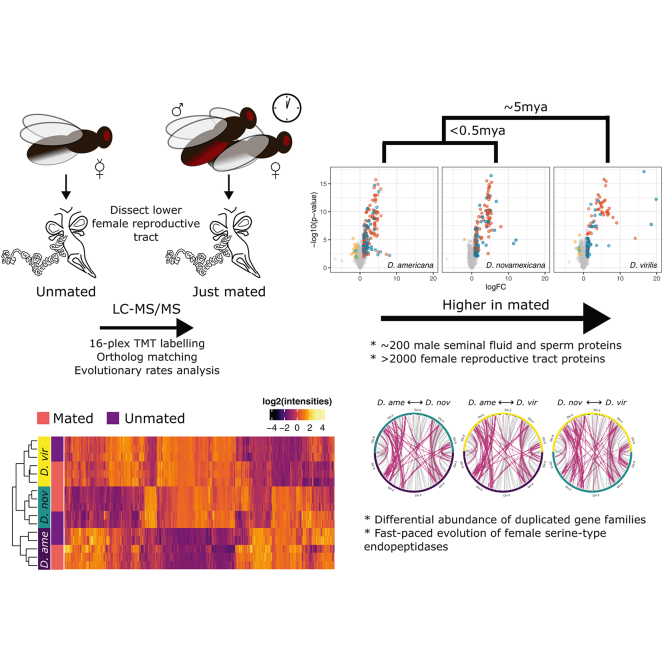

## In Brief

Molecular interactions between the sexes are critical for fertilization success. However, coevolution of interacting reproductive traits can lead to biochemical mismatches between populations and the emergence of postmating prezygotic isolation. Here, we identify and quantify the transferred male ejaculate proteome and female reproductive proteome using three species of the *virilis* group. We identified over 200 male ejaculate proteins and over 2000 female reproductive tract proteins, many of which show differential abundance between species, including female-specific serine-type endopeptidases.

Molecular interactions between gametes and the accompanying reproductive fluid are critical for fertilization success (1, 2, 3, 4, 5, 6). In internally fertilizing taxa, additional complexity arises compared with external fertilizers because sperm must traverse the female reproductive tract. Moreover, frequent polyandry and extended periods of sperm storage can present the opportunity for postcopulatory sexual selection (7, 8, 9) and the need to maintain sperm viability and retention in storage (10). Conflicts over reproduction that arise from the differing reproductive interests of the sexes can also lead to the evolution of female and male strategies to control reproduction (11, 12). Such female × male interactions may be population or species specific, which can result in rapid codiversification of reproductive traits within populations (1, 13, 14).

Codiversification of ejaculate × female reproductive tract interactions within populations may result in biochemical mismatches between populations and lead to the emergence of postmating prezygotic isolation (14, 15). Postmating prezygotic isolation is thought to be a rapidly evolving and important barrier to gene flow early during speciation and is often observed between recently diverged species or populations that are undergoing incipient speciation (16, 17, 18, 19, 20, 21, 22, 23). Postmating prezygotic isolation acts after mating but before fertilization by preventing successful fertilizations in heterospecific crosses between taxa (14, 15). A heterospecific male ejaculate may fare worse in postcopulatory interactions (24, 25), have reduced sperm motility (26, 27), or elicit an abnormal postmating female response, which can cause impaired sperm use or reduced oviposition rates (16, 28, 29, 30, 31). A growing body of literature has begun to characterize the molecular differences between taxa that contribute to postmating prezygotic isolation (15, 28, 29, 31, 32, 33, 34, 35, 36).

The female reproductive tract and the male ejaculate contain a mixture of carbohydrates, lipids, microbes, and proteins (37, 38, 39). However, we still lack a comprehensive understanding of how the composition of this reproductive fluid influences fertilization success. Proteins in the female and male reproductive fluid (*i.e.*, the *reproductive proteome*) can have important impacts on fertilization. In *Drosophila melanogaster*, around 300 male seminal fluid proteins (SFPs) have been identified, some of which are needed to trigger the postmating female response that affects female postmating morphology, physiology, and behavior (6, 40). Female-derived proteins are also critical in determining fertilization success (3, 26, 28, 41, 42, 43, 44, 45). Yet, the molecular composition and function of the female reproductive tract proteome in most model systems has received far less attention compared with the male, despite requisite interactions between female and male molecules (28, 29, 46, 47, 48, 49, 50, 51). Differences between species in the composition of the female and/or male reproductive proteome likely contribute to postmating prezygotic isolation (15). For instance, males may transfer a mixture of SFPs during mating that differ in abundance or identity between species (33) that elicits an abnormal postmating female response in a heterospecific female (31). Likewise, the female reproductive tract environment may express a combination of proteins that interact with the male ejaculate in a species-specific manner that is disrupted after heterospecific mating (28).

Species in the *Drosophila virilis* clade provide an ideal system in which to characterize divergence in the male ejaculate and female reproductive tract proteomes. Postmating prezygotic isolation is pervasive in laboratory crosses between species in the *virilis* group, where heterospecific sperms are rapidly lost from the female sperm storage organs (seminal receptacle and spermathecae) or become incapacitated, resulting in dramatically reduced interspecific fertilization success (18, 30, 52). Previous studies in the *virilis* group have shown that many SFPs evolve rapidly, species show divergent gene expression patterns in the male reproductive tract (36), and several rapidly evolving SFPs lie within paternal quantitative trait loci that are associated with paternal postmating prezygotic incompatibility (36). Furthermore, gene expression in the female reproductive tract is significantly perturbed after heterospecific mating (29). Therefore, it is likely that postmating prezygotic isolation is, in part, the result of mismatched ejaculate × female reproductive tract interactions that are caused by species differences in the composition of the female and male reproductive proteomes. Thus, efficiently identifying and characterizing the complement of potentially interacting male and female proteins in key model systems is essential for advancing our understanding of reproductive evolution.

To identify male ejaculate proteins (SFPs and sperm proteins) that are transferred during mating requires distinguishing proteins that are transferred to the female reproductive tract from other so-called “housekeeping” proteins found in the reproductive tract tissues. One successful approach is to use heavy isotopic labeling of one sex and to distinguish mass spectra by a mass:charge shift. The first extensive list of transferred male SFPs in *Drosophila* was achieved by rearing flies of one sex on heavy isotope–labeled media (53, 54). While this approach has been the gold standard for identifying protein origin in a mixture of two or more samples, this method is time consuming, labour intensive, and may need optimization for different species. Other approaches have recently been applied; for example, Sepil *et al.* (55) identified over 50 novel male *D. melanogaster* SFPs by comparing the abundance of proteins in the accessory glands and ejaculatory ducts—the main source of male SFPs in insects (56)—of males before and after mating (55).

**Fig. 1:**
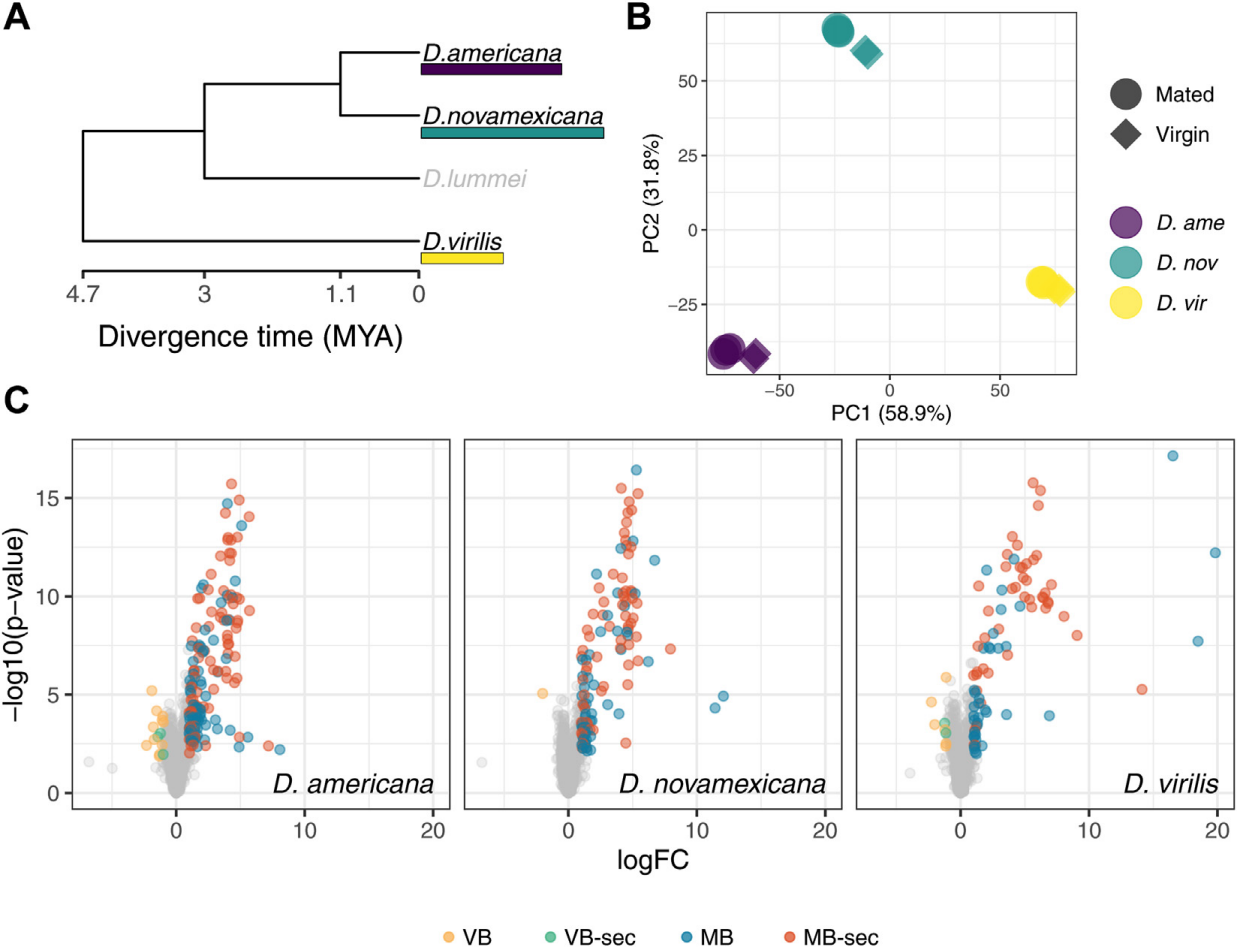
**Overview and identification of ejaculate proteins.***A*, phylogenetic relationship between species in the *virilis* group. Estimated divergence times (million year ago, MYA) from Yusuf *et al.* (105). *B*, principal component analysis (PCA) plot for the 500 most variable proteins across all samples. Together, PC1 and PC2 explained over 90% of the variance. *C*, volcano plots demonstrating identification of ejaculate proteins in each species. Positive values indicate higher abundance in mated samples. Colored points show proteins higher abundance (log2-fold change > |1| and FDR corrected *p* value < 0.05) in mated samples with (MB-sec) or without a signal peptide (MB) or virgin samples with (VB-sec) or without a signal peptide (VB). FDR, false discovery rate.

In this study, we present a simple quantitative method to simultaneously characterize the female reproductive tract proteome and the transferred male ejaculate proteome while avoiding the need for heavy isotope labeling. We performed LC–MS/MS analysis using tandem mass tag (TMT)-labeled samples to compare protein abundances in the lower reproductive tracts of unmated females to those that were collected immediately after mating (thus containing ejaculate proteins) for three species in the *virilis* group: the sister species *Drosophila americana* and *Drosophila* *novamexicana* and the more distantly related *D. virilis* (Fig. 1*A*). Copulation duration in the *virilis* group is ∼2 min, and the majority of postmating female responses in Diptera emerge 3 to 6 h after mating (28, 29, 49, 57, 58). Few changes in gene expression occur immediately or shortly after mating (58, 59), such that we expect few changes in protein abundance in the female reproductive tract contributed by the female shortly after mating allowing us to identify mostly male ejaculate contributions.

Overall, we predict greater divergence in protein abundance between species for male- compared with female-derived proteins as male reproductive proteins evolve faster than female proteins on average (29). Furthermore, we expect secreted proteins, such as proteins containing a predicted signal peptide sequence, will be overrepresented in the set of differentially abundant proteins between species, as secreted proteins often act as signaling molecules between the sexes to influence fertilization success. Finally, we expect fewer differences between the sister species *D. americana* and *D. novamexicana* compared with comparisons involving the more distantly related *D. virilis*. We found that over half of ejaculate proteins showed differential abundance between species compared with less than one-third of female reproductive tract proteins we identified. Similar numbers of ejaculate proteins showed differential abundance between species in all comparisons, whereas for female reproductive tract proteins, we found the fewest differences between *D. novamexicana* and *D. virilis*. Male ejaculate proteins containing a signal peptide sequence showed elevated rates of molecular evolution, as expected. However, these predicted secretory proteins were not more likely to show differences in abundance between species, contrary to our expectations. Finally, female-specific serine-type endopeptidases were evolving as fast as male SFP candidates.

## Experimental Procedures

### Fly Stocks and Maintenance

The following iso-female lines were used for each species: *D. americana* SB.02.06 (obtained from Bryant F. McAllister, University of Iowa), *D. novamexicana* 15010-1031.04, and *D. virilis* 15010-1051.49 (National Drosophila Species Stock Center, Cornell University). Flies were raised on cornmeal/sucrose media supplemented with yeast and kept at 22 °C on a natural light cycle. We collected virgins within 2 days of eclosion and kept flies in single sex groups of 10 to 25 until sexually mature (∼10 days old).

### Tissue Collection and Protein Extraction

We paired virgin flies of the same species individually in food vials and allowed a 3 h opportunity to mate. We flash froze flies in liquid nitrogen within 30 s after copulation—which lasts ∼2 min—terminated and stored flies at −80 °C until dissections. All copulations were observed. For virgin samples, females were processed in the same way but never exposed to males. We thawed females and dissected the lower reproductive tract (uterus, seminal receptacle, and spermathecae) in a drop of PBS using fine forceps. We pooled 40 to 50 female reproductive tracts per replicate in 50 μl lysis buffer (5% sodium deoxycholate; 1% SDS in HPLC grade water), kept on ice, and then freeze thawed pooled samples (×3) by placing on dry ice for 5 min, thawing to room temperature, and then vortexing for 30 s. Finally, we centrifuged samples at 17,000*g* for 5 min at 4 °C and collected the resulting supernatant. Samples were stored at −80 °C and shipped to the Cambridge Centre for Proteomics on dry ice for further processing.

### Liquid Chromatography–Tandem Mass Spectrometry

LC–MS/MS was performed at the Cambridge Centre for Proteomics using ThermoFisher Scientific TMTpro 16plex Isobaric Label Reagent Set 0.5 mg. Labeling was performed according to the manufacturer's instructions. About 80 μg of proteins per sample was labeled, and protein estimation was done using RC-DC protein assay from Bio-Rad.

Labeled samples were cleaned on SepPack C18 cartridge from Waters before being fractionated on ACQUITY UPLC system using ACQUITY UPLC BEH C18 1.7 μm, 2.1 × 150 mm column. Parameters of the chromatography method are as follows: flow 0.244 ml/min, linear gradient starting from 95% of buffer A: 20 mM ammonium formate pH 10, 5% buffer B: 20 mM ammonium formate in 80% acetonitrile pH 10 ending at 100% of buffer B during the time of 75 min. PDA detector lamp setting: 210 nm to 400 nm 1 min fractions were collected starting from peptide elution observed on chromatogram. Fractions were concatenated: first fraction with the middle run fraction and so on to achieve a different elution profile per combined fraction later when performing LC–MS/MS. In total, 15 fractions were produced for LC–MS/MS. Dried fractions from the high pH reverse-phase separations were resuspended in 30 μl of 0.1% formic acid and placed into a glass vial. About 1 μl of each fraction was injected by the HPLC autosampler and separated by the LC method detailed later. Fractions were combined into pairs (*i.e.*, the first fraction with the middle fraction etc.) and were analyzed by LC–MS/MS.

LC–MS/MS experiments were performed using a Dionex Ultimate 3000 RSLC nanoUPLC (Thermo Fisher Scientific, Inc) system and a Lumos Orbitrap mass spectrometer (Thermo Fisher Scientific, Inc). Peptides were loaded onto a precolumn (Thermo Scientific PepMap 100 C18, 5 mm particle size, 100 Å pore size, 300 mm i.d. × 5 mm length) from the Ultimate 3000 autosampler with 0.1% formic acid for 3 min at a flow rate of 10 μl/min. After this period, the column valve was switched to allow elution of peptides from the precolumn onto the analytical column. Separation of peptides was performed by C18 reverse-phase chromatography at a flow rate of 300 nl/min using a Thermo Scientific reverse-phase nano Easy-spray column (Thermo Scientific PepMap C18, 2 mm particle size, 100 Å pore size, 75 mm i.d. × 50 cm length). Solvent A was water + 0.1% formic acid, and solvent B was 80% acetonitrile, 20% water + 0.1% formic acid. The linear gradient employed was 2 to 40% B in 93 min. Total LC run time was 120 min including a high organic wash step and column re-equilibration.

The eluted peptides from the C18 column LC eluant were sprayed into the mass spectrometer by means of an Easy-Spray source (Thermo Fisher Scientific, Inc). All *m/z* values of eluting peptide ions were measured in an Orbitrap mass analyzer, set at a resolution of 120,000, and were scanned between *m/z* 380 and 1500 Da. Data-dependent MS/MS scans (Top Speed) were employed to automatically isolate and fragment precursor ions by collision-induced dissociation (normalized collision energy: 35%), which were analyzed in the linear ion trap. Singly charged ions and ions with unassigned charge states were excluded from being selected for MS/MS, and a dynamic exclusion window of 70 s was employed. The top ten most abundant fragment ions from each MS/MS event were then selected for a further stage of fragmentation by synchronous precursor celection MS3 (1) in the high-energy collisional dissociation high-energy collision cell using high-energy collisional dissociation (normalized collision energy: 65%). The *m/z* values and relative abundances of each reporter ion and all fragments (mass range from 100 to 500 Da) in each MS3 step were measured in the Orbitrap analyzer, which was set at a resolution of 60,000. This was performed in cycles of ten MS3 events before the Lumos instrument reverted to scanning the *m/z* ratios of the intact peptide ions and the cycle continued.

### Protein Identification and Quantitation

We first generated a custom reference proteome database for each species. Briefly, we mapped available RNA-Seq data from male reproductive tissues of the three species to their respective genomes using HISAT2, version 2.2.1 (60) and assembled their transcriptomes using StringTie, version 2.1.5 (61). For *D. novamexicana* and *D. virilis*, we used the recently updated National Center for Biotechnology Information RefSeq assembly and gene annotations (GCF_003285875.2 and GCF_003285735.1, respectively) as a guide in transcriptome assembly, and we used a recently generated NanoPore genome of *D. americana* (strain SB02.06; PRJNA923584). Assembled transcripts were annotated, and protein coding genes were predicted with TransDecoder (https://github.com/TransDecoder). We used the longest isoform of each protein-coding gene in downstream database searches and analyses, where we searched the RAW mass spectral data iteratively against each species complete proteome.

RAW spectral files were first converted to mzML format using msConvert (62) and subsequently analyzed using the Trans-Proteomic Pipeline (TPP), version 6.1.0 (63). Protein database searches were performed using X!Tandem (64) and Comet (65) using decoys to establish peptide probability modeling. We specified trypsin as enzyme with a maximum of two missed cleavages, 16-plex TMTpro and carbamidomethyl (C) as fixed modifications, and oxidation (M) and deamidation (NQ) as variable modifications. Protein identification allowed an MS tolerance of ±10 ppm and an MS/MS tolerance of ±0.8 Da ppm along with permission of up to two missed tryptic cleavages. Peptide spectral matches and protein assignments were validated with PeptideProphet and ProteinProphet, respectively. Quantification was performed using Libra on the 16 reporter ion intensities per peptide by calculating the sum of centroided reporter ions within a ±2 millimass unit window. Protein abundances were calculated as the summed intensities for all associated peptides after filtering. The implementation pipeline and the specific parameter files for the TPP analysis are available on GitHub (https://github.com/YazBraimah/TPP_TMT_proteomics_pipeline). Mass spectrometry data are available *via* the ProteomeXchange Consortium (ID: PXD031638).

### Ortholog Identification and Combined Database Compilation

We identified 1:1:1 reciprocal orthologs between *D. americana*, *D. novamexicana*, and *D. virilis* using OrthoFinder (66, 67), with the three species complete proteome sequence files as input and default settings. The total number of protein isoforms used in the analysis was as follows: *D. americana* (11,958 entries), *D. novamexicana* (12,729 entries), and *D. virilis* (12,792 entries). We identified 10,777 protein “orthogroups,” the majority corresponding to a single protein for each species (73%–77%; supplemental Fig. S1*A*). However, over 23% of proteins belong to an orthogroup that contains more than one protein, that is, representing gene families. The *D. americana* proteome contains more proteins belonging to multigene families (supplemental Fig. S1*B*).

After searching the RAW mass spectra against each species proteome to obtain protein quantitation for our samples, we combined species-specific abundance data for each protein by orthogroups. This resulted in some proteins being duplicated in the dataset, that is, there was one-to-many or many-to-one orthology between species (supplemental Fig. S2, *A* and *B*). To this combined dataset, we added a curated list of orthology assignments based on syntenic BLAST that we identified using custom scripts (https://github.com/YazBraimah/BlastWithSynteny) and that were not assigned to orthogroups using OrthoFinder. The combined database comprised 2132 unique protein orthogroups, of which 117 orthogroups represented more than one protein (supplemental Fig. S2, *A* and *B*).

The protein quantitation information compiled using each species proteome as reference allowed us to assess the utility of alternate- *versus* species-specific query databases for protein identification and quantitation. In the Appendix, we show that species-specific protein quantitation provides a more accurate and sensitive approach for detecting differentially abundant proteins between species. Based on our results, we caution against using a single species reference proteome for comparative evolutionary proteomics using data-dependent acquisition.

### Experimental Design and Statistical Rationale

In total, we collected 16 samples for labeling using the 16-plex TMT reagent kit, which enables robust quantitative comparisons of multiplexed samples run simultaneously, thus eliminating batch effects (68). We collected three biological replicates for mated samples for each species and *D. virilis* virgin samples and two replicates each for *D. americana* and *D. novamexicana* virgin samples. We chose this design to increase replication of the virgin *D. virilis* samples, which we expect to have diverged most from the sister species *D. americana* and *D. novamexicana*. We also expect greater differences between mated samples because of the rapid evolution of male SFPs.

We applied stringent thresholds for peptide (false discovery rate [FDR] < 0.01) and protein identification (FDR < 0.05) and ensured accuracy of quantitative estimates using strict mass tolerance and retention time cutoffs. We performed differential abundance analyses between samples using the edgeR package (69) in R, version 4.2.2 (70) (see Statistical Analysis section). For differential abundance analyses, we filtered proteins identified by two or more unique peptides and normalized abundance data using the trimmed mean of M-values method. Differences in protein abundance were determined based on a log2 fold change >|1| and FDR-corrected *p*-value <0.05. We inspected diagnostic plots for all analyses and corrected *p*-values for multiple testing using the Benjamini–Hochberg method (71).

### Statistical Analysis

We performed all statistical analyses in R, version 4.2.2 (70). Complete code and analysis can be found on GitHub (https://martingarlovsky.github.io/virilisProteomics/). We performed principal component (PC) analyses using the *prcomp* function for the 500 most variable proteins across all samples. For differential abundance analyses, we filtered proteins identified by fewer than two unique peptides and used *voom* normalization and fitted protein-wise linear models (*lmFit*) with empirical Bayes smoothing (*eBayes*) using edgeR (69).

### Evolutionary Rate Analysis

We obtained previously published (29) pairwise rates of nonsynonymous (dN) to synonymous (dS) nucleotide substitutions (dN/dS; ω) calculated using PAML (phylogenetic analysis by maximum likelihood) (72).

### Gene Ontology Analysis

We performed Gene Ontology (GO) enrichment analysis using Trinotate GO annotations with the topGO package (73). We used the entire proteome as background to perform enrichment tests using Fisher’s exact method, with a minimum of 15 genes per term. All GO terms reported had an FDR-corrected *p*-value <0.05.

### Signal Peptide Identification

To identify potentially secreted proteins, we queried each species entire proteome using Phobius (https://phobius.sbc.su.se/) (74) and SignalP-5.0 (75) to identify proteins containing a predicted signal peptide sequence and combined the resulting lists for each species. This *in silico* approach is conservative as other secretory pathways are possible. However, we also note that inclusion of a signal peptide sequence is not synonymous with a secretory function.

## Results

We identified 3014 (supplemental Table S1), 3156 (supplemental Table S2), and 3149 (supplemental Table S3) proteins using the *D. americana*, *D. novamexicana*, and *D. virilis* query databases, respectively, and only retained proteins with two or more unique peptides (Table 1). To identify ejaculate proteins (*i.e.*, putative SFPs and sperm proteins), we first performed differential abundance analysis between mated and virgin samples for each species, using each species database. Most ejaculate proteins identified in each species had orthologs in another species (77–85%). For the remaining ejaculate proteins, we assigned orthology using a custom syntenic BLAST pipeline (see *Experimental procedures* section), after which only 9 (4%), 29 (11%), and 8 (3%) ejaculate proteins remained unassigned to orthogroups in *D. americana*, *D. novamexicana*, and *D. virilis*, respectively (Table 1). Whether these proteins are truly unique in each species requires further investigation.

**Table 1:** Summary of numbers of proteins identified using each species database and the combined database (CDB)

	Database			
	*Drosophila americana*	*Drosophila novamexicana*	*Drosophila virilis*	CDB
Total no. of proteins	3014	3156	3149	2132
Two unique peptides	2963	3125	3093	2128
Ejaculate candidates	257	262	230	223
Ejaculate candidates (unique)	37	70	64	NA
Ejaculate divergence				
*D.americana versus D. novamexicana*	39	42	38	140
*D. americana versus D. virilis*	101	99	72	148
*D. novamexicana versus D. virilis*	128	117	78	132
Female reproductive tract divergence				
*D. americana versus D. novamexicana*	119	118	116	624
*D. americana versus D. virilis*	266	275	257	672
*D. novamexicana versus D. virilis*	306	319	296	405

Numbers of differentially abundant proteins between species for ejaculate proteins and virgin female reproductive tracts. For the combined database, the number of proteins identified by two unique peptides was calculated using at least one search database.

Abbreviation: NA, not available.

The combined database comprised 2132 unique protein orthogroups (supplemental Table S4). This accounts for ∼68% to 71% of proteins identified in our samples using each species query database, which is similar to the percentage of all proteins in the proteome assigned to orthogroups using OrthoFinder (∼72%–78%). The majority of proteins in the combined database (95%; 2015/2132) belong to a single orthogroup, having 1:1:1 orthology between all three species. However, the remaining orthogroups (n = 117) showed one-to-many or many-to-one orthology between species (supplemental Fig. S2*B*). We report results where we analyzed duplicated genes separately from the single copy orthologs unless otherwise stated.

A PC analysis showed that the first three components explain more than 97% of the variance. PC1 (58.9% of variance explained) separated samples by species and mating treatment, with the largest difference between species, followed by mating treatment (Fig. 1*B*). PC2 and PC3 explained 31.8% and 6.4% of the variance, respectively, further separating virgin from mated samples (Fig. 1*B*). Most proteins showed no difference in abundance between virgin and mated samples. We identified orthologs of 17% (193/1108) of the *D. melanogaster* sperm proteome, of which over 90% (175/193) showed no difference in abundance between mated and virgin samples. We also identified 48 female reproductive tract–biased genes (based on previous mRNA expression profiling) and 93 proteins that were previously found to show significant changes in gene expression after mating (29); only 12 proteins showed differences in abundance between virgin *versus* mated samples. We excluded these 12 proteins from our list of ejaculate proteins (see later). No proteins with serine-type endopeptidase annotation were more abundant in virgin samples, suggesting that the female reproductive tract proteome had not yet undergone significant postmating changes in our samples—as expected.

### Male Ejaculate Proteome

We identified 229 proteins (belonging to 223 orthogroups) that were significantly more abundant in mated samples using the combined database (henceforth “ejaculate proteins”; Fig. 1*C*). Over one-third of ejaculate proteins identified were shared between all three species (n = 59; 34%–66%) (supplemental Fig. S3). More than half of ejaculate proteins (>52%) had a signal peptide sequence, indicative of a protein being secreted (Fig. 1*C*).

Ejaculate proteins included orthologs of 27 *D. melanogaster* SFPs (40), including two members of the Sex Peptide network; Aquarius (*CG14061*), and *CG9997* (76); and orthologs of 19 *D. melanogaster* sperm proteins (77, 78). In addition, 41 proteins overlap with genes that were inferred to be SFPs by mRNA expression or otherwise showed accessory gland or ejaculatory bulb–biased expression in the *virilis* group (supplemental Fig. S4; (36)). Ejaculate proteins were enriched for GO terms that are commonly associated with sperm proteins and SFPs, including metabolic and catabolic processes, sperm individualization, sperm mitochondrion organization, and sperm storage (biological process [BP]), extracellular region and extracellular exosome (cellular component [CC]), enzyme regulator activity, and molecule binding (molecular function [MF]) (supplemental Table S5).

It has been suggested that transferred male ejaculate proteins may be “swamped out” by the more abundant proteins in the female reproductive tract (55). The average abundance of ejaculate proteins was lower than the remaining (female reproductive tract) proteome in all three species (Mann–Whitney *U* test, all *p* < 0.004) (supplemental Fig. S5).

### Ejaculate Divergence

**Fig. 2:**
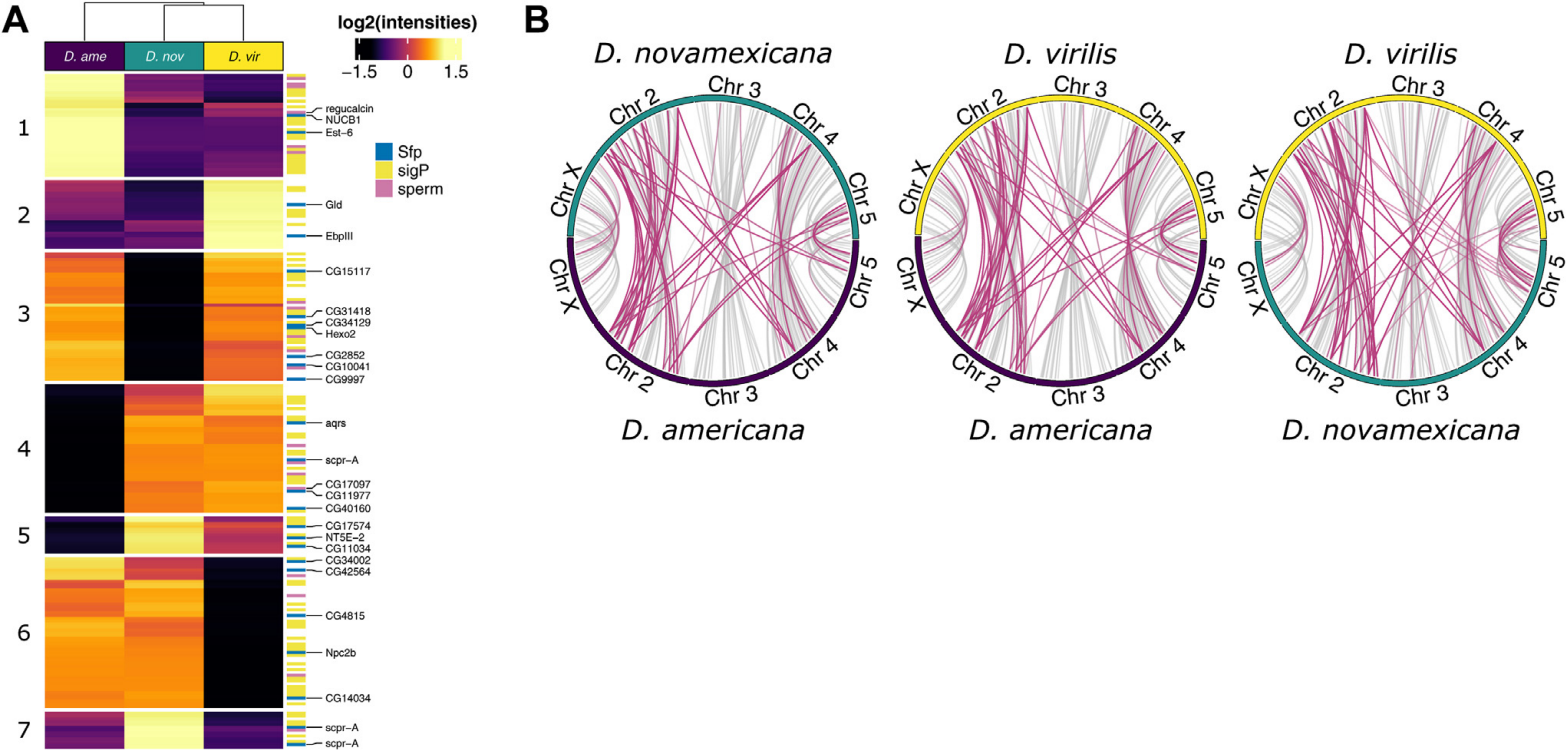
**Ejaculate protein divergence.***A*, heatmap of ejaculate protein abundances (n = 229). Values are median centred log2(normalized abundances) averaged across biological replicates. K-means clusters (k = 7) are indicated on the *left*. Signal peptides and *Drosophila melanogaster* sperm and SFP orthologs are highlighted on the *right*. *D. melanogaster* SFP orthologs are named on the *right*. *B*, pairwise synteny plots for duplicated ejaculate proteins between species on each of the five major chromosomes in the *virilis* group. All genes shown in *light gray*, and genes belonging to multigene families are overlaid in *dark pink*. SFP, seminal fluid protein.

To identify differences in the transferred male ejaculate proteome between species, we performed differential abundance analyses between each species pair using all ejaculate proteins (n = 229, including gene duplicates). There were similar numbers of differentially abundant proteins between species in all comparisons (n = 132–148, or 58%–65%; Table 1 and supplemental Table S4). Differentially abundant ejaculate proteins included orthologs of 17 *D. melanogaster* SFP and 15 sperm proteins (supplemental Table S6). Proteins with predicted signal peptides were not more or less likely to be differentially abundant between species in any comparison (Fisher's exact tests, all *p* > 0.13). Similarly, *D. melanogaster* sperm proteins were equally likely to show differential abundance in all comparisons (Fisher's exact tests, all *p* > 0.47; Fig. 2*A*). Differentially abundant ejaculate proteins again showed GO enrichment for metabolic processes, sperm storage, sperm individualization (BP); extracellular region and extracellular exosome (CC); and serine-type endopeptidase inhibitor activity (MF), among others (supplemental Table S7).

Using *k*-means clustering, we identified seven clusters of proteins, of which five distinguished between species in terms of protein abundance (Fig. 2*A* and supplemental Fig. S6). Clusters contained proteins more or less abundant in one or more species compared with the other(s). Of note, we identified two orthologs of proteins that differed in abundance between species that are major components of the mating plug in *D. melanogaster*, EbpIII and Est-6. EbpIII was more abundant in *D. virilis* compared with both *D. americana* and *D. novamexicana*, whereas Est-6 was more abundant in *D. americana* relative to the other two species. Orthologs of two members of the *D. melanogaster* Sex Peptide network (Aquarius and CG9997) also showed significant differences in abundance between species (Fig. 2*A*). These results show that SFP abundance—which is not often considered when analysing SFP function—differs between species and may confer functional consequences affecting fertility.

Interestingly, all 31 ejaculate proteins belonging to duplicated gene families were differentially abundant between species in at least one comparison. There were fewer ejaculate proteins belonging to gene families than expected by chance (*p* = 0.023, proportion of 99,999 simulated draws greater than or equal to the observed number sampled without replacement) (supplemental Fig. S7), suggesting these 31 proteins are not a random subset of the entire proteome. We also examined the distribution of SFP orthologs along the five major chromosomes and find that the majority of duplicated SFPs between species are derived from single copy clusters on chromosome 2 (Fig. 2*B*), which was previously shown to have a significant overrepresentation of SFP genes in this species group (36).

### Female Reproductive Tract Proteome

**Fig. 3:**
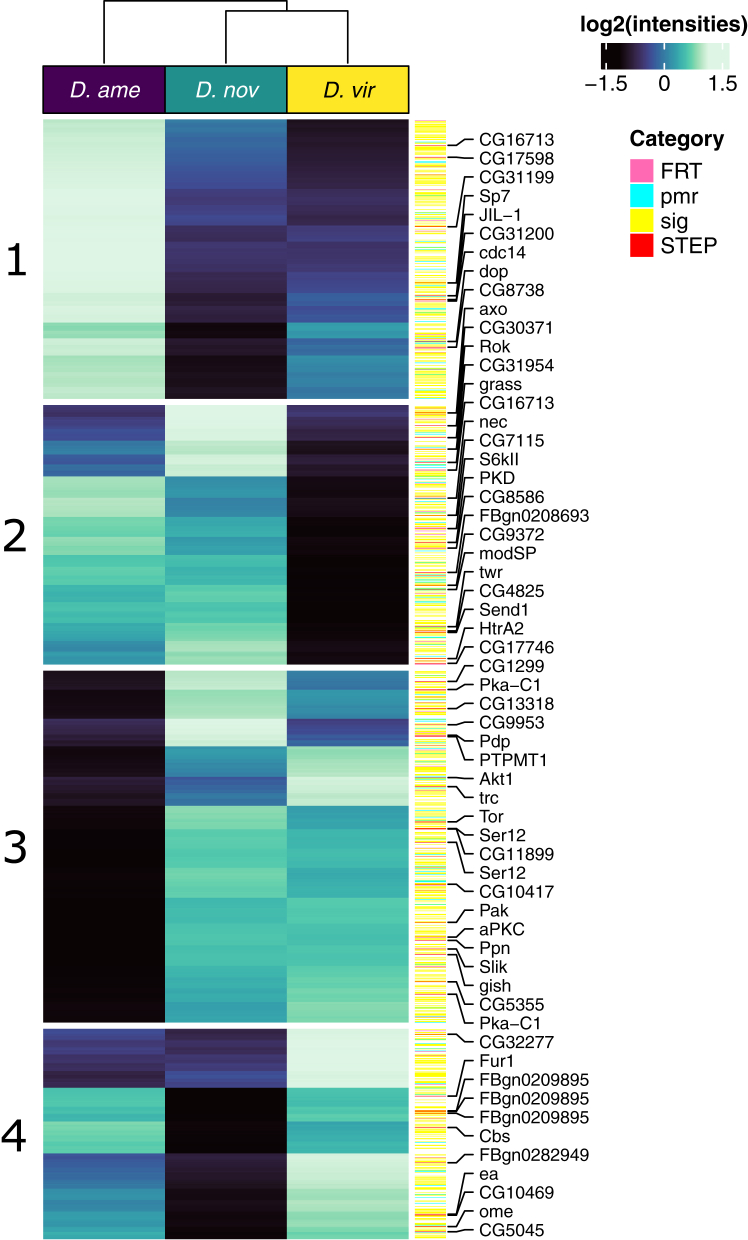
**Female reproductive tract (FRT) proteome (n = 2313).** K-means clusters (k = 4) are shown on the *left*. Female reproductive tract–biased genes (FRT; *pink*), postmating response genes (pmr; *light blue*), signal peptides (sig; *yellow*), and serine-type endopeptidases (STEP; *red*) are highlighted on the *right*. Values are median centred log2(normalized abundances) averaged across biological replicates.

After excluding transferred male ejaculate proteins, the female reproductive tract proteome consisted of 2313 proteins belonging to 1909 orthogroups (Fig. 3). Over 41% (805/1949) of female reproductive tract proteins we identified contained a predicted signal peptide sequence (supplemental Table S9); proteins likely secreted into the reproductive tract lumen and thus may directly interact with the male ejaculate. Overall, female reproductive tract proteins showed GO enrichment for peptide and protein transport, endocytosis and exocytosis, translation, cellular organization, vesicle fusion, peptide and protein secretion (BP), cytoplasm, cytosol, extracellular exosome, and various vesicle and membrane terms (CC), many molecule-binding terms, and enzyme activity (MF) (supplemental Table S8). Twenty-two proteins were more abundant in virgin compared with mated samples. However, no GO terms reached statistical significance (*p* < 0.05) for these proteins. It is also worth noting that many of these female proteins are likely involved in basic cellular processes and are not directly involved in reproductive processes and interactions, which highlights a limitation of our approach in interrogating female reproductive proteins.

### Female Reproductive Tract Proteome Divergence

We performed differential abundance analysis between virgin samples after excluding ejaculate proteins (n = 2313, including gene duplicates). There were fewer differentially abundant proteins between *D. novamexicana* and *D. virilis* (405; 21%) than between *D. americana* and *D. novamexicana* (624; 32%) or between *D. americana* and *D. virilis* (672; 34%) (Table 1). Signal peptides (n = 805) were not more likely to be differentially abundant between species in all comparisons (Fisher’s exact test, all *p* > 0.15) (Fig. 3). Differentially abundant female reproductive tract proteins showed GO enrichment for various molecule transport terms, muscle contraction and development, neurogenesis, and wound healing (BP), cytoplasm and membranes (CC), and various molecule-binding terms (MF) (supplemental Table S9). Similar to ejaculate proteins, over 97% (889/914) of proteins belonging to gene families showed differential abundance between species in at least one comparison.

Using *k*-means clustering, we identified four clusters of proteins differentiating between species (Fig. 3 and supplemental Fig. S8). As with male ejaculate proteins, each cluster contained proteins more or less abundant in one or more species compared with the other(s). Of note, many proteins previously identified as characteristic of the postmating female response, female reproductive tract–biased genes, and serine-type endopeptidases showed differences in abundance between species (Fig. 3 and supplemental Fig. S8).

### Evolutionary Rates

**Fig. 4:**
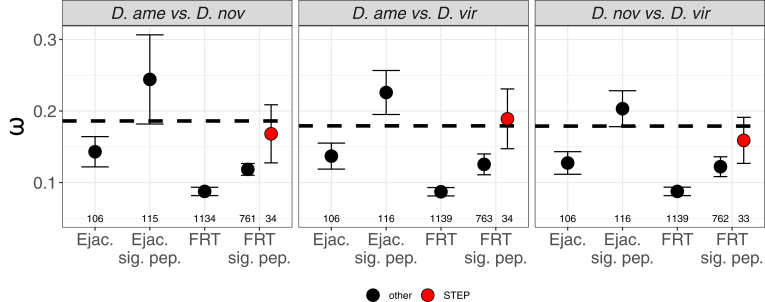
**Evolutionary ratesof reproductive proteins.** Pairwise nonsynonymous to synonymous substitution rates (ω; mean ± standard error) for ejaculate proteins and female reproductive tract proteins with or without a signal peptide sequence. Female reproductive tract proteins with a signal peptide sequence and serine-type endopeptidase (STEP) annotation are shown in *red*. Numbers below points indicate number of genes. *Dashed line* represents the genome average ω in each comparison (*Drosophila americana versus Drosophila novamexicana* = [mean ± SE] 0.186 ± 0.003, n = 13,158; *D. americana versus Drosophila virilis* = 0.179 ± 0.003, n = 13,319; *D. novamexicana versus D. virilis* = 0.179 ± 0.003, n = 13,307).

Ejaculate proteins with a signal peptide sequence showed elevated rates of molecular evolution (dN/dS ratios) compared with the remaining ejaculate proteins between *D. americana* and *D. virilis* (Mann–Whitney *U* test, *p* = 0.016) and between *D. novamexicana* and *D. virilis* (*p* = 0.010) but not between *D. americana* and *D. novamexicana* (*p* = 0.226). Ejaculate proteins with a signal peptide were also evolving faster than the genome average between *D. americana* and *D. virilis* (*p* = 0.039) but not between *D. americana* and *D. novamexicana* (*p* = 0.617) or *D. novamexicana* and *D. virilis* (*p* = 0.124) (Fig. 4). Ejaculate proteins with a signal peptide and serine-type endopeptidase annotation evolved at a similar rate to other proteins with signal peptides (*p* > 0.062 in all comparisons). The remaining ejaculate proteins were evolving at a similar rate to the genome average in all comparisons (all *p* > 0.063; Fig. 4).

Female reproductive tract proteins with a signal peptide sequence were evolving faster than the remaining female reproductive tract proteins (*p* < 0.001 in all comparisons) but more slowly than the genome average (*p* < 0.001 in all comparisons; Fig. 4). Female reproductive tract proteins with serine-type endopeptidase annotation and a signal peptide sequence showed elevated dN/dS compared with other secreted female reproductive tract proteins (*p* < 0.008 in all comparisons) and in all comparisons were evolving as fast as ejaculate proteins (*p* > 0.569 in all comparisons; Fig. 4). These secreted female reproductive tract proteins were also evolving at a similar rate to the genome average (all *p* > 0.546).

### Correlation Between mRNA and Protein Abundance of Transferred Ejaculate Proteins

Previous work has often found scant evidence for a correlation between mRNA and protein abundance (79, 80, 81). Here, we explored the relationship between mRNA abundance in male tissues (obtained from (36)) and protein abundance of transferred ejaculate proteins and find varying correlations depending on the tissue examined (supplemental Fig. S9). The highest Pearson correlations were found between mRNA transcripts that are expressed in the accessory glands or ejaculatory bulb and ejaculate proteins with a secretion signal (*R* = 0.25–0.6 in all species), and these correlations are significant in all three species (all *p* < 0.011; supplemental Fig. S10). These results indicate that while the correlations between mRNA and protein abundance are weak, some tissues show significantly higher correlations than others and that these tissues likely represent the source of transferred ejaculate proteins.

## Discussion

To understand the molecular interactions that mediate fertilization success and postmating prezygotic isolation requires identifying differentiation in the reproductive proteome of both mating partners. Here, we simultaneously characterized the male ejaculate and female reproductive tract proteome for three species in the *virilis* group. We identified a number of differentially abundant proteins between species in both the female and male reproductive proteomes. In crosses between species, these species differences in protein abundance may cause mismatched ejaculate × female reproductive tract interactions. Female serine-type endopeptidases were evolving as fast as male SPFs, implicating rapid diversification of this class of female-derived proteins in postcopulatory interactions and potential emergence of postmating prezygotic isolation.

We identified over 200 candidate ejaculate proteins (SFPs and sperm proteins), including previously identified putative SFPs from the *virilis* group, and orthologs of *D. melanogaster* SFPs and sperm proteins. Recall that in crosses between *virilis* group species, sperm are rapidly lost from the female sperm storage organs or become incapacitated (30). Several differentially abundant proteins between species we identified are notable for being orthologs of *D. melanogaster* SFPs with known effects on sperm entry and exit from storage in *D. melanogaster* (Fig. 2*A*). Ejaculatory bulb protein III (FBgn0011695) is integral to formation of the posterior mating plug, which affects female sperm storage and fertility (82). Glucose dehydrogenase (FBgn0001112) affects sperm uptake and release from the spermathecae (83). Although we did not detect Sex Peptide, we did identify two other members of the Sex Peptide network. Sex Peptide induces the long-term postmating female response in *D. melanogaster* and requires interactions among various members of the Sex Peptide network for binding of Sex Peptide to sperm (76). Together, our findings suggest that males transfer species-specific abundances of SFPs during copulation. As such, females may not process a heterospecific ejaculate effectively, or receipt of an abnormal cocktail of SFPs may elicit an abnormal postmating female response (29).

The molecular composition of the female reproductive tract has historically not received the same level of interrogation as the male ejaculate. Our analysis expands on a growing effort to characterize the female reproductive tract proteome (28, 43, 48, 49, 50, 51, 57, 84). Interestingly, more than 90% of *D. melanogaster* sperm protein orthologs we identified showed no difference in abundance between mated *versus* virgin samples, suggesting substantive expression in the female reproductive tract (85). Even though our approach captures all proteins that are expressed and detectable in the female reproductive tract (*i.e.*, does not “enrich” for reproduction-related proteins), we are still able to identify a significant number of likely candidates. Specifically, we identified over 800 female reproductive tract proteins that include a signal peptide sequence, thus may be secreted into the reproductive tract lumen. Accordingly, secreted female reproductive tract proteins were enriched for GO terms relating to molecule binding, proteolysis, and enzyme activity including proteins with peptidase activity, which act to cleave other proteins (see later) (45, 86, 87). These proteins provide interesting candidates to investigate as they may interact with—and play a role in processing—the male ejaculate.

Male SFPs are predicted to play a disproportionate role in postmating prezygotic isolation because of their rapid diversification compared with proteins expressed in the female reproductive tract (29). This is evident in the much greater proportion of differentially abundant ejaculate proteins between species (more than 57% of ejaculate proteins showed differential abundance between species), compared with the more modest proportions of proteins differing in abundance between female reproductive tract proteomes (∼21%–32%). Unsurprisingly, however, we did identify a host of differentially abundant female reproductive tract proteins with orthologs in *D. melanogaster* linked to fertility. For instance, Send1 (FBgn0031406) is expressed in the spermathecae and recruits sperm into storage and reduces sperm motility in the seminal receptacle (88). Immune-induced molecule 33 (FBgn0031561) is secreted into the hemolymph upon microbial infection. Likewise, modSP (FBgn0051217) is involved in the immune response. The potential role of microbes transferred during mating to postmating prezygotic isolation (39) or nevertheless that females mount an immune response against a heterospecific ejaculate requires further investigation.

Female reproductive tract genes evolve more conservatively than male reproductive genes and the genome average in the *virilis* group (29). However, we found that female serine-type endopeptidases evolved at an elevated rate, similar to that of male SFPs. Serine-type endopeptidases mediate the cleavage of SFPs in the female reproductive tract that are required to properly stimulate the postmating female response (45, 86, 87). We also found that serine-type endopeptidases and their inhibitors were prominent among differentially abundant ejaculate proteins. In the cabbage white butterfly (*Pieris rapae*) and ants (*Atta colombica*), females secrete proteases that digest the male ejaculate, possibly to control sperm use (84, 89, 90). In turn, the male ejaculate may contain protease inhibitors to counteract such female control (91). Sequence and abundance divergence between species of endopeptidases and their inhibitors has been implicated in postmating prezygotic isolation across a range of taxa (28, 33, 84, 89, 92). Further analyses using divergence and polymorphism data are required to determine the evolutionary forces contributing to the molecular evolution of male- and female-derived proteins that contribute to postmating prezygotic isolation (93, 94, 95, 96).

Identifying transferred male ejaculate proteins has long presented a challenge for research investigating reproductive proteins. Comparing mated *versus* virgin samples allowed us to identify putative SFPs without the need for labor-intensive labeling techniques. Some of the differentially abundant proteins between mated and virgin samples may have been contributed by the female rather than transferred by the male, induced by courtship or during mating. However, few proteins previously identified as characteristic of the postmating female response or showing female reproductive tract bias (29) showed differences in abundance, consistent with the finding that the majority of postmating female responses in *Drosophila* take place ∼3 h postmating (29, 59). Further studies are necessary to establish the molecular changes involved in any “pericopulatory” female response.

## Data Availability

All code and analysis are available on GitHub: https://martingarlovsky.github.io/virilisProteomics/. The mass spectrometry proteomics data have been deposited to the ProteomeXchange Consortium *via* the PRIDE (97) partner repository with the dataset identifier PXD031638.

## Supplemental data

This article contains supplemental data (98, 99, 100, 101, 102, 103, 104).

## Conflict of interest

The authors declare no competing interests.

## Supplemental Data

Figure A1

Figure A2

Figure A3

Figure S1 **Orthofinder results for entire proteomes.** The majority or orthogroups contain a single protein. Note the difference in y-axis scales between single copy vs. multi-copy genes.

Figure S2 **Representation of proteins identified in the current study belonging to orthogroups.***A*, number of proteins belonging to single copy or multi-copy gene families. Note the axis break on the y-axis to improve clarity of the number of multi-copy genes. *B*, relative copy number position between species for proteins belonging to a gene family in one or more species (n = 117). Lines show copy number relationship for each protein between species.

Figure S3 **Shared and unique ejaculate proteins between species, including those without orthologs, using each species database**.

Figure S4 **Overlap between proteins identified in the current study (Prot) with accessory gland (AG), ejaculatory bulb (EB), or SFPs identified by Ahmed-Braimah *et al.*** (36).

Figure S5 **Abundance of ejaculate proteins vs. remaining female reproductive tract (FRT) proteins in each species.** Points are individual proteins. *P*-values obtained from Mann-Whitney U tests.

Figure S6 **Abundance of ejaculate proteins in each k-means cluster (k = 7), 5 of which distinguished groups of proteins differing in abundance between species.** Shown are normalised abundances for ejaculate proteins in each cluster. Large white points show the mean and small points are individual proteins. Thick and thin black bars showing the 66% and 95% confidence intervals, respectively. *P*-values indicate results from post-hoc Tukey's honest significant difference tests after analysis of variance performed on each cluster; ns: non-significant; ∗*p* < 0.05; ∗∗*p* < 0.01; ∗∗∗*p* < 0.001.

Figure S7 **Density plot for permutation test sampling random draws without replacement of single copy or duplicate proteins from the entire proteome.***P*-value calculated as the total number of simulated draws (no. draws = 99,999) greater than or equal to the observed number of proteins belonging to multi-copy gene families (n = 31).

Figure S8 **Abundance of female reproductive tract proteins in each cluster.** K-means clustering was used to separate proteins into clusters (k = 4), which distinguished groups of proteins differing in abundance between species. White points show the mean with thick and thin black bars showing the 66% and 95% confidence intervals, respectively. *p*-values represent results from post-hoc Tukey's honest significant difference tests after analysis of variance performed on each cluster; ns: non-significant; ∗*p* < 0.05; ∗∗*p* < 0.01; ∗∗∗*p* < 0.001.

Figure S9 **Correlation between mRNA abundance and protein abundance for ejaculate proteins across the three species and four male tissues: accessory glands, ejaculatory bulb, testes, and gonadectomized carcass.** Correlation coefficients and *p*-values are indicated within each panel and separately for ejaculate proteins and ejaculate proteins with a predicted signal peptide.

## References

[1] Pitnick S., Wolfner M.F., Suarez S.S. (2009). Sperm Biology: An Evolutionary Perspective.

[2] Vacquier V.D., Swanson W.J. (2011). Selection in the rapid evolution of gamete recognition proteins in marine invertebrates. Cold Spring Harb. Perspect. Biol..

[3] Johnson S.L., Borziak K., Kleffmann T., Rosengrave P., Dorus S., Gemmell N.J. (2020). Ovarian fluid proteome variation associates with sperm swimming speed in an externally fertilizing fish. J. Evol. Biol..

[4] Wainwright S.M., Hopkins B.R., Mendes C.C., Sekar A., Kroeger B., Hellberg J.E.E.U. (2021). *Drosophila* sex peptide controls the assembly of lipid microcarriers in seminal fluid. Proc. Natl. Acad. Sci. U. S. A..

[5] Karr T.L., Swanson W.J., Snook R.R., Birkhead T.R., Hosken D.J., Pitnick S. (2009). Sperm Biology [Internet].

[6] Wolfner M.F. (2009). Battle and ballet: molecular interactions between the sexes in *Drosophila*. J. Hered..

[7] Firman R.C., Gasparini C., Manier M.K., Pizzari T. (2017). Postmating female control: 20 years of cryptic female choice. Trends Ecol. Evol..

[8] Birkhead T.R., Pizzari T. (2002). Postcopulatory sexual selection. Nat. Rev. Genet..

[9] Taylor M.L., Price T.A.R., Wedell N. (2014). Polyandry in nature: a global analysis. Trends Ecol. Evol..

[10] Liberti J., Baer B., Boomsma J.J. (2016). Queen reproductive tract secretions enhance sperm motility in ants. Biol. Lett..

[11] Parker G.A. (1979). Sexual Selection and Reproductive Competition in Insects [Internet].

[12] Eberhard W.G. (1996).

[13] Wensing K.U., Fricke C. (2018). Divergence in sex peptide-mediated female post-mating responses in *Drosophila melanogaster*. Proc. R. Soc. B Biol. Sci..

[14] Howard D.J., Palumbi S.R., Birge L.M., Manier M.K. (2009). Sperm Biology: An Evolutionary Perspective.

[15] McDonough C.E., Whittington E., Pitnick S., Dorus S. (2016). Proteomics of reproductive systems: towards a molecular understanding of postmating, prezygotic reproductive barriers. J. Proteomics.

[16] Turissini D.A., McGirr J.A., Patel S.S., David J.R., Matute D.R. (2018). The rate of evolution of postmating-prezygotic reproductive isolation in *Drosophila*. Mol. Biol. Evol..

[17] Jennings J.H., Snook R.R., Hoikkala A. (2014). Reproductive isolation among allopatric *Drosophila montana* populations. Evolution.

[18] Ahmed-Braimah Y.H., McAllister B.F. (2012). Rapid evolution of assortative fertilisation between recently allopatric species of *Drosophila*. Int. J. Evol. Biol..

[19] Devigili A., Fitzpatrick J.L., Gasparini C., Ramnarine I.W., Pilastro A., Evans J.P. (2018). Possible glimpses into early speciation: the effect of ovarian fluid on sperm velocity accords with post-copulatory isolation between two guppy populations. J. Evol. Biol..

[20] Dopman E.B., Robbins P.S., Seaman A. (2010). Components of reproductive isolation between North American pheromone strains of the European corn borer. Evolution.

[21] Fricke C., Arnqvist G. (2004). Divergence in replicated phylogenies: the evolution of partial post-mating prezygotic isolation in bean weevils. J. Evol. Biol..

[22] Hewitt G.M., Mason P., Nichols R.A. (1989). Sperm precedence and homogamy across a hybrid zone in the alpine grasshopper *Podisma pedestris*. Heredity.

[23] Gregory P.G., Howard D.J. (1994). A postinsemination barrier to fertilization isolates two closely related ground crickets. Evolution.

[24] Manier M.K., Lüpold S., Belote J.M., Starmer W.T., Berben K.S., Ala-Honkola O. (2013). Postcopulatory sexual selection generates speciation phenotypes in *Drosophila*. Curr. Biol..

[25] Price C.S.C. (1997). Conspecific sperm precedence in *Drosophila*. Nature.

[26] Cramer E.R.A., Ålund M., McFarlane S.E., Johnsen A., Qvarnström A. (2016). Females discriminate against heterospecific sperm in a natural hybrid zone. Evolution.

[27] Yeates S.E., Diamond S.E., Einum S., Emerson B.C., Holt W.V., Gage M.J.G. (2013). Cryptic choice of conspecific sperm controlled by the impact of ovarian fluid on sperm swimming behaviour. Evolution.

[28] McCullough E., McDonough C., Pitnick S., Dorus S. (2020). Quantitative proteomics reveals rapid divergence in the postmating response of female reproductive tracts among sibling species. Proc. R. Soc. B Biol. Sci..

[29] Ahmed-Braimah Y.H., Wolfner M.F., Clark A.G. (2021). Differences in postmating transcriptional responses between conspecific and heterospecific matings in *Drosophila*. Mol. Biol. Evol..

[30] Ahmed-Braimah Y.H. (2016). Multiple genes cause postmating prezygotic reproductive isolation in the *Drosophila virilis* group. G3 (Bethesda).

[31] Diaz F., Allan C.W., Chen X., Coleman J.M., Bono J.M., Matzkin L.M. (2022). Divergent evolutionary trajectories shape the postmating transcriptional profiles of conspecifically and heterospecifically mated cactophilic Drosophila females. Commun. Biol..

[32] Garlovsky M.D., Yusuf L.H., Ritchie M.G., Snook R.R. (2020). Within-population sperm competition intensity does not predict asymmetry in conpopulation sperm precedence. Philos. Trans. R. Soc. B Biol. Sci..

[33] Garlovsky M.D., Evans C., Rosenow M.A., Karr T.L., Snook R.R. (2020). Seminal fluid protein divergence among populations exhibiting postmating prezygotic reproductive isolation. Mol. Ecol..

[34] Rowe M., Whittington E., Borziak K., Ravinet M., Eroukhmanoff F., Sætre G.P. (2019). Molecular diversification of the seminal fluid proteome in a recently diverged Passerine species pair. Mol. Biol. Evol..

[35] Nakadera Y., Smith A.T., Daupagne L., Coutellec M.A., Koene J.M., Ramm S.A. (2020). Divergence of seminal fluid gene expression and function among natural snail populations. J. Evol. Biol..

[36] Ahmed-Braimah Y.H., Unckless R.L., Clark A.G. (2017). Evolutionary dynamics of male reproductive genes in the *Drosophila virilis* subgroup. G3 (Bethesda).

[37] Scolari F., Khamis F.M., Pérez-Staples D. (2021). Beyond sperm and male accessory gland proteins: exploring insect reproductive metabolomes. Front. Physiol..

[38] Perry J.C., Sirot L., Wigby S. (2013). The seminal symphony: how to compose an ejaculate. Trends Ecol. Evol..

[39] Rowe M., Veerus L., Trosvik P., Buckling A., Pizzari T. (2020). The reproductive microbiome: an emerging driver of sexual selection, sexual conflict, mating systems, and reproductive isolation. Trends Ecol. Evol..

[40] Wigby S., Brown N.C., Allen S.E., Misra S., Sitnik J.L., Sepil I. (2020). The *Drosophila* seminal proteome and its role in postcopulatory sexual selection. Philos. Trans. R. Soc. B Biol. Sci..

[41] Gasparini C., Pilastro A., Evans J.P. (2020). The role of female reproductive fluid in sperm competition. Philos. Trans. R. Soc. B Biol. Sci..

[42] Fitzpatrick J.L., Willis C., Devigili A., Young A., Carroll M., Hunter H.R. (2020). Chemical signals from eggs facilitate cryptic female choice in humans. Proc. R. Soc. B Biol. Sci..

[43] Keeble S., Firman R.C., Sarver B.A.J., Clark N.L., Simmons L.W., Dean M.D. (2021). Evolutionary, proteomic, and experimental investigations suggest the extracellular matrix of cumulus cells mediates fertilization outcomes. Biol. Reprod..

[44] Avila F.W., Mattei A.L., Wolfner M.F. (2015). Sex peptide receptor is required for the release of stored sperm by mated *Drosophila melanogaster* females. J. Insect Physiol..

[45] LaFlamme B.A., Avila F.W., Michalski K., Wolfner M.F. (2014). A *Drosophila* protease cascade member, seminal metalloprotease-1, is activated stepwise by male factors and requires female factors for full activity. Genetics.

[46] McDonough-Goldstein C.E., Pitnick S., Dorus S. (2021). *Drosophila* oocyte proteome composition covaries with female mating status. Sci. Rep..

[47] McDonough-Goldstein C.E., Borziak K., Pitnick S., Dorus S. (2021). *Drosophila* female reproductive tract gene expression reveals coordinated mating responses and rapidly evolving tissue-specific genes. G3 (Bethesda).

[48] Fazeli A., Affara N.A., Hubank M., Holt W.V. (2004). Sperm-induced modification of the oviductal gene expression profile after natural insemination in mice. Biol. Reprod..

[49] Mack P.D., Kapelnikov A., Heifetz Y., Bender M. (2006). Mating-responsive genes in reproductive tissues of female Drosophila melanogaster. Proc. Natl. Acad. Sci. U. S. A..

[50] Swanson W.J., Wong A., Wolfner M.F., Aquadro C.F. (2004). Evolutionary expressed sequence tag analysis of *Drosophila* female reproductive tracts identifies genes subjected to positive selection. Genetics.

[51] Kapelnikov A., Zelinger E., Gottlieb Y., Rhrissorrakrai K., Gunsalus K.C., Heifetz Y. (2008). Mating induces an immune response and developmental switch in the Drosophila oviduct. Proc. Natl. Acad. Sci. U. S. A..

[52] Sweigart A.L. (2010). The genetics of postmating, prezygotic reproductive isolation between *Drosophila virilis* and *D. americana*. Genetics.

[53] Findlay G.D., Yi X., MacCoss M.J., Swanson W.J. (2008). Proteomics reveals novel *Drosophila* seminal fluid proteins transferred at mating. PLoS Biol..

[54] Findlay G.D., MacCoss M.J., Swanson W.J. (2009). Proteomic discovery of previously unannotated, rapidly evolving seminal fluid genes in *Drosophila*. Genome Res..

[55] Sepil I., Hopkins B.R., Dean R., Thézénas M.L., Charles P.D., Konietzny R. (2019). Quantitative proteomics identification of seminal fluid proteins in male *Drosophila melanogaster*. Mol. Cell. Proteomics.

[56] Avila F.W., Sirot L.K., LaFlamme B.A., Rubinstein C.D., Wolfner M.F. (2011). Insect seminal fluid proteins: identification and function. Annu. Rev. Entomol..

[57] McDonough-Goldstein C.E., Whittington E., McCullough E.L., Buel S.M., Erdman S., Pitnick S. (2021). Pronounced postmating response in the *Drosophila* female reproductive tract fluid proteome. Mol. Cell. Proteomics.

[58] Alfonso-Parra C., Ahmed-Braimah Y.H., Degner E.C., Avila F.W., Villarreal S.M., Pleiss J.A. (2016). Mating-induced transcriptome changes in the reproductive tract of female *Aedes aegypti*. PLoS Negl. Trop. Dis..

[59] Bono J.M., Matzkin L.M., Kelleher E.S., Markow T.A. (2011). Postmating transcriptional changes in reproductive tracts of con- and heterospecifically mated *Drosophila mojavensis* females. Proc. Natl. Acad. Sci. U. S. A..

[60] Kim D., Paggi J.M., Park C., Bennett C., Salzberg S.L. (2019). Graph-based genome alignment and genotyping with HISAT2 and HISAT-genotype. Nat. Biotechnol..

[61] Pertea M., Pertea G.M., Antonescu C.M., Chang T.C., Mendell J.T., Salzberg S.L. (2015). StringTie enables improved reconstruction of a transcriptome from RNA-seq reads. Nat. Biotechnol..

[62] Adusumilli R., Mallick P., Comai L., Katz J.E., Mallick P. (2017). Proteomics: Methods and Protocols [Internet].

[63] Deutsch E.W., Mendoza L., Shteynberg D., Slagel J., Sun Z., Moritz R.L. (2015). Trans-Proteomic pipeline, a standardized data processing pipeline for large-scale reproducible proteomics informatics. Proteomics Clin. Appl..

[64] Craig R., Beavis R.C. (2004). TANDEM: matching proteins with tandem mass spectra. Bioinformatics.

[65] Eng J.K., Jahan T.A., Hoopmann M.R. (2013). Comet: an open-source MS/MS sequence database search tool. Proteomics.

[66] Emms D.M., Kelly S. (2015). OrthoFinder: solving fundamental biases in whole genome comparisons dramatically improves orthogroup inference accuracy. Genome Biol..

[67] Emms D.M., Kelly S. (2019). OrthoFinder: phylogenetic orthology inference for comparative genomics. Genome Biol..

[68] O'Connell J.D., Paulo J.A., O'Brien J.J., Gygi S.P. (2018). Proteome-wide evaluation of two common protein quantification methods. J. Proteome Res..

[69] Robinson M.D., McCarthy D.J., Smyth G.K. (2010). edgeR: a bioconductor package for differential expression analysis of digital gene expression data. Bioinformatics.

[70] R Core Team (2022).

[71] Benjamini Y., Hochberg Y. (1995). Controlling the false discovery rate: a practical and powerful approach to multiple testing. J. R. Stat. Soc. Ser. B Methodol..

[72] Yang Z. (2007). PAML 4: phylogenetic analysis by maximum likelihood. Mol. Biol. Evol..

[73] Alexa A., Rahnenfuhrer J. (2022).

[74] Käll L., Krogh A., Sonnhammer E.L.L. (2004). A combined transmembrane topology and signal peptide prediction method. J. Mol. Biol..

[75] Petersen T.N., Brunak S., von Heijne G., Nielsen H. (2011). SignalP 4.0: discriminating signal peptides from transmembrane regions. Nat. Methods.

[76] Singh A., Buehner N.A., Lin H., Baranowski K.J., Findlay G.D., Wolfner M.F. (2018). Long-term interaction between *Drosophila* sperm and sex peptide is mediated by other seminal proteins that bind only transiently to sperm. Insect Biochem. Mol. Biol..

[77] Dorus S., Busby S.A., Gerike U., Shabanowitz J., Hunt D.F., Karr T.L. (2006). Genomic and functional evolution of the *Drosophila melanogaster* sperm proteome. Nat. Genet..

[78] Wasbrough E.R., Dorus S., Hester S., Howard-Murkin J., Lilley K., Wilkin E. (2010). The *Drosophila melanogaster* sperm proteome-II (DmSP-II). J. Proteomics.

[79] Liu Y., Beyer A., Aebersold R. (2016). On the dependency of cellular protein levels on mRNA abundance. Cell.

[80] Harnik Y., Buchauer L., Ben-Moshe S., Averbukh I., Levin Y., Savidor A. (2021). Spatial discordances between mRNAs and proteins in the intestinal epithelium. Nat. Metab..

[81] Buccitelli C., Selbach M. (2020). mRNAs, proteins and the emerging principles of gene expression control. Nat. Rev. Genet..

[82] Avila F.W., Cohen A.B., Ameerudeen F.S., Duneau D., Suresh S., Mattei A.L. (2015). Retention of ejaculate by *Drosophila melanogaster* females requires the male-derived mating plug protein PEBme. Genetics.

[83] Iida K., Cavener D.R. (2004). Glucose dehydrogenase is required for normal sperm storage and utilization in female Drosophila melanogaster. J. Exp. Biol..

[84] Plakke M.S., Walker J.L., Lombardo J.B., Goetz B.J., Pacella G.N., Durrant J.D. (2019). Characterization of female reproductive proteases in a butterfly from functional and evolutionary perspectives. Physiol. Biochem. Zool..

[85] McCullough E.L., Whittington E., Singh A., Pitnick S., Wolfner M.F., Dorus S. (2022). The life history of *Drosophila* sperm involves molecular continuity between male and female reproductive tracts. Proc. Natl. Acad. Sci. U. S. A..

[86] Kelleher E.S., Pennington J.E. (2009). Protease gene duplication and proteolytic activity in Drosophila female reproductive tracts. Mol. Biol. Evol..

[87] Avila F.W., Wolfner M.F. (2017). Cleavage of the *Drosophila* seminal protein Acp36DE in mated females enhances its sperm storage activity. J. Insect Physiol..

[88] Schnakenberg S.L., Matias W.R., Siegal M.L. (2011). Sperm-storage defects and live birth in Drosophila females lacking spermathecal secretory cells. PLoS Biol..

[89] Meslin C., Cherwin T.S., Plakke M.S., Small B.S., Goetz B.J., Morehouse N.I. (2017). Structural complexity and molecular heterogeneity of a butterfly ejaculate reflect a complex history of selection. Proc. Natl. Acad. Sci. U. S. A..

[90] Dosselli R., Grassl J., den Boer S.P.A., Kratz M., Moran J.M., Boomsma J.J. (2019). Protein-level interactions as mediators of sexual conflict in ants∗. Mol. Cell. Proteomics.

[91] LaFlamme B.A., Wolfner M.F. (2013). Identification and function of proteolysis regulators in seminal fluid. Mol. Reprod. Dev..

[92] Al-Wathiqui N., Lewis S.M., Dopman E.B. (2018). Molecular dissection of nuptial gifts in divergent strains of Ostrinia moths. Physiol. Entomol..

[93] Dapper A.L., Wade M.J. (2020). Relaxed selection and the rapid evolution of reproductive genes. Trends Genet..

[94] Patlar B., Jayaswal V., Ranz J.M., Civetta A. (2021). Non-adaptive molecular evolution of seminal fluid proteins in Drosophila. Evolution.

[95] Gutiérrez-Valencia J., Fracassetti M., Horvath R., Laenen B., Désamore A., Drouzas A.D. (2022). Genomic signatures of sexual selection on pollen-expressed genes in Arabis alpina. Mol. Biol. Evol..

[96] Wiberg R.A.W., Brand J.N., Schärer L. (2021). Faster rates of molecular sequence evolution in reproduction-related genes and in species with hypodermic sperm morphologies. Mol. Biol. Evol..

[97] Perez-Riverol Y., Bai J., Bandla C., García-Seisdedos D., Hewapathirana S., Kamatchinathan S. (2022). The PRIDE database resources in 2022: a hub for mass spectrometry-based proteomics evidences. Nucleic Acids Res..

[98] Swanson W.J., Vacquier V.D. (2002). The rapid evolution of reproductive proteins. Nat. Rev. Genet..

[99] Stockley P., Franco C., Claydon A.J., Davidson A., Hammond D.E., Brownridge P.J. (2020). Revealing mechanisms of mating plug function under sexual selection. Proc. Natl. Acad. Sci. U. S. A..

[100] Degner J.F., Marioni J.C., Pai A.A., Pickrell J.K., Nkadori E., Gilad Y. (2009). Effect of read-mapping biases on detecting allele-specific expression from RNA-sequencing data. Bioinformatics.

[101] Vicens A., Borziak K., Karr T.L., Roldan E.R.S., Dorus S. (2017). Comparative sperm proteomics in mouse species with divergent mating systems. Mol. Biol. Evol..

[102] Bayram H.L., Claydon A.J., Brownridge P.J., Hurst J.L., Mileham A., Stockley P. (2016). Cross-species proteomics in analysis of mammalian sperm proteins. J. Proteomics.

[103] Whittington E., Forsythe D., Borziak K., Karr T.L., Walters J.R., Dorus S. (2017). Contrasting patterns of evolutionary constraint and novelty revealed by comparative sperm proteomic analysis in Lepidoptera. BMC Genomics.

[104] Garlovsky M.D., Sandler J.A., Karr T.L. (2022). Functional diversity and evolution of the Drosophila sperm proteome. Mol. Cell. Proteomics.

[105] Yusuf L.H., Tyukmaeva V., Hoikkala A., Ritchie M.G. (2022). Divergence and introgression among the virilis group of Drosophila. Evol. Lett..

