# Supplementary figures and images for: Evolutionary Quantitative Proteomics of Reproductive Protein Divergence in Drosophila
Source: Mol Cell Proteomics. 2023 Jun 28;22(8):100610. doi: 10.1016/j.mcpro.2023.100610 (PMC10407754; doi:10.1016/j.mcpro.2023.100610)

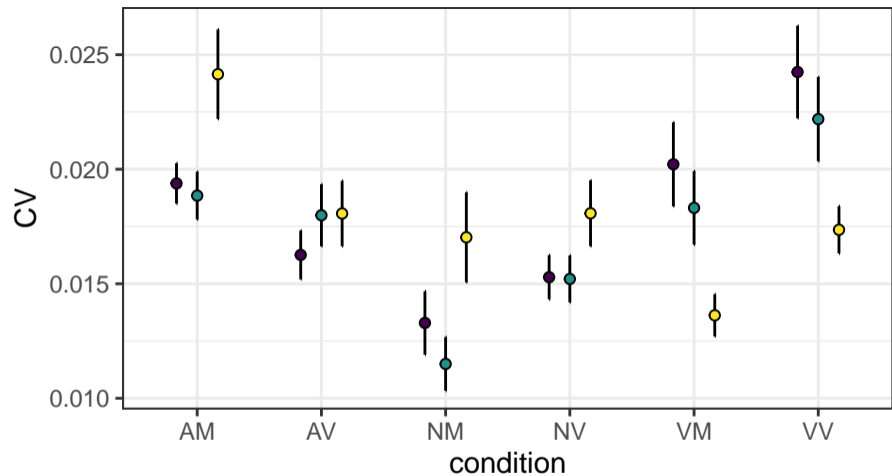

Database: ● *D. ame* ● *D. nov* ● *D. vir*

Supplement: Figure A1 [file mmc1.pdf]

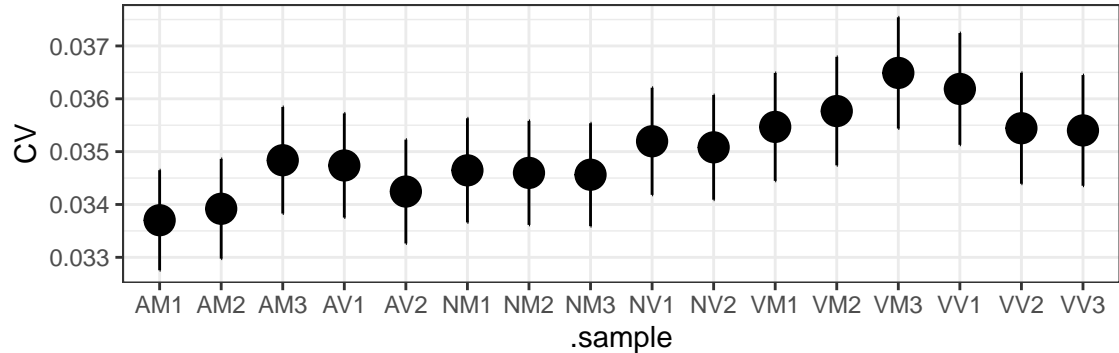

Supplement: Figure A2 [file mmc2.pdf]

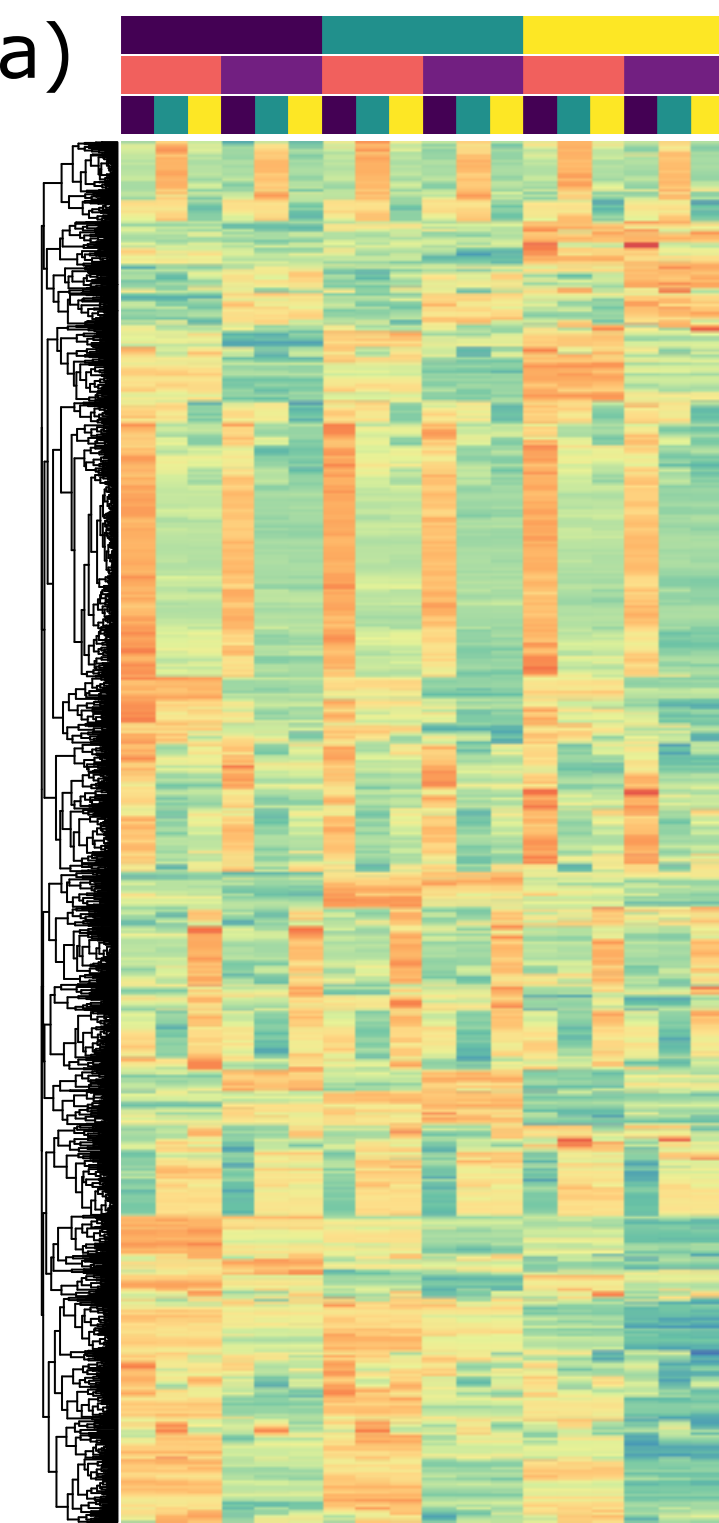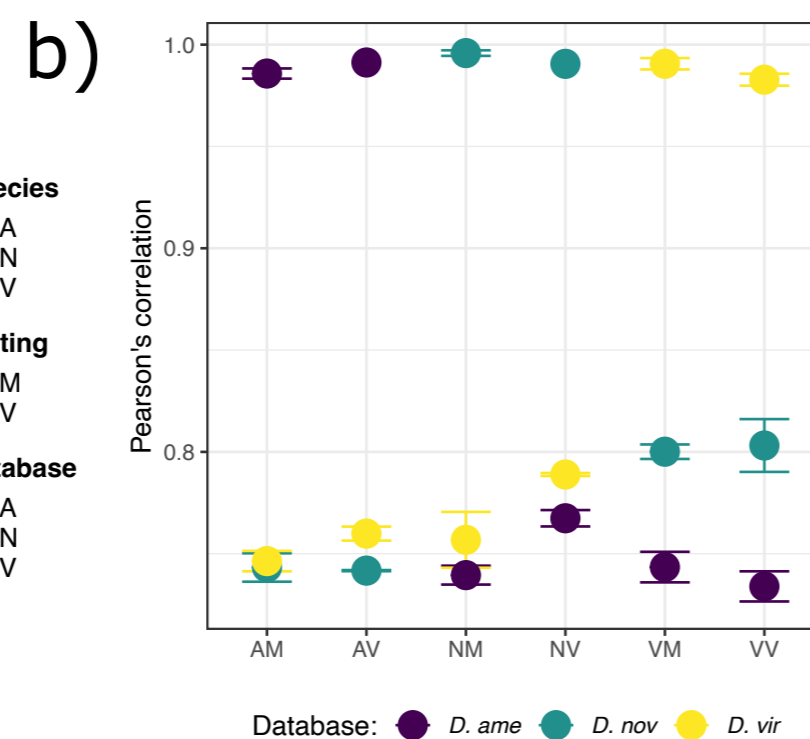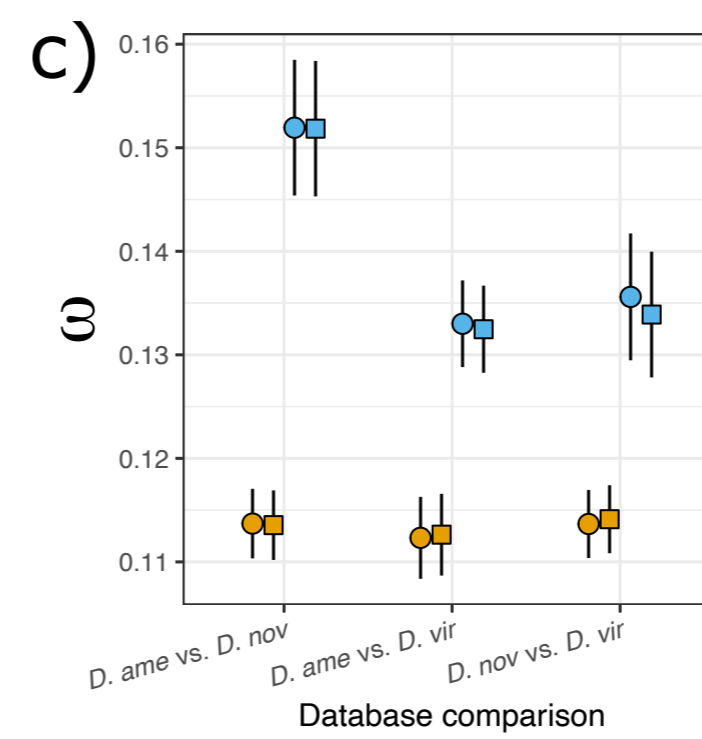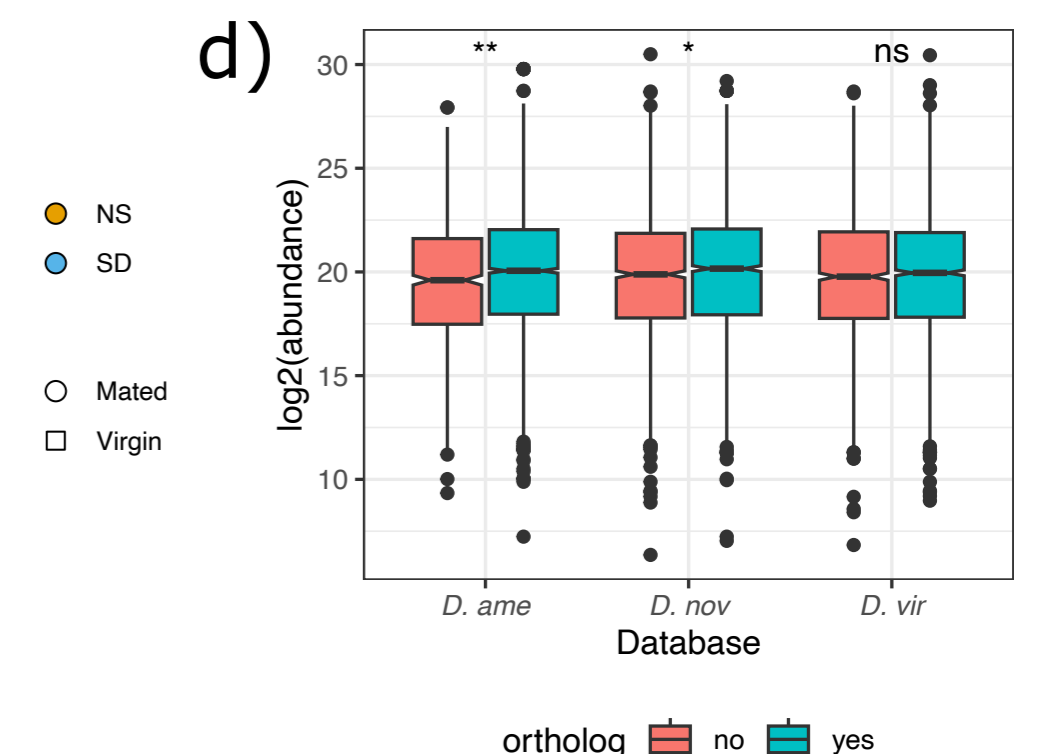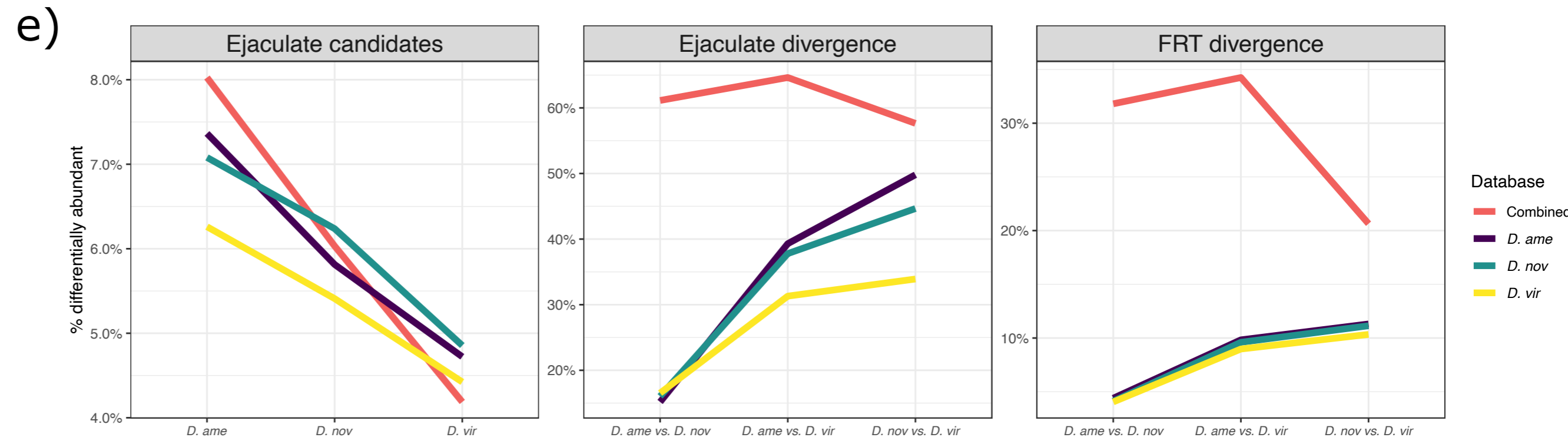

Supplement: Figure A3 [file mmc3.pdf]

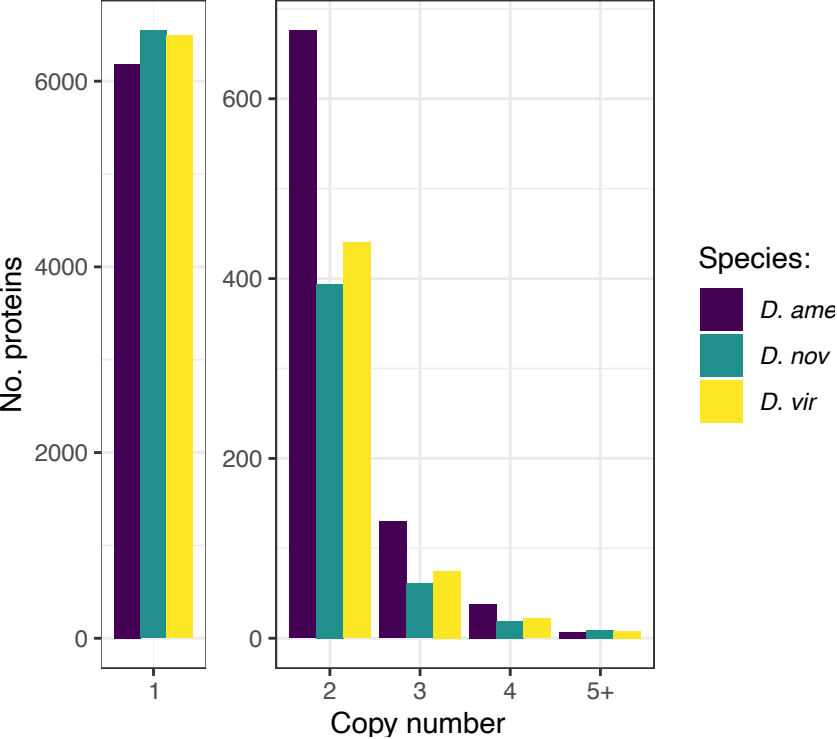

Supplement: Figure S1 — Orthofinder results for entire proteomes. The majority or orthogroups contain a single protein. Note the difference in y-axis scales between single copy vs. multi-copy genes. [file mmc4.pdf]

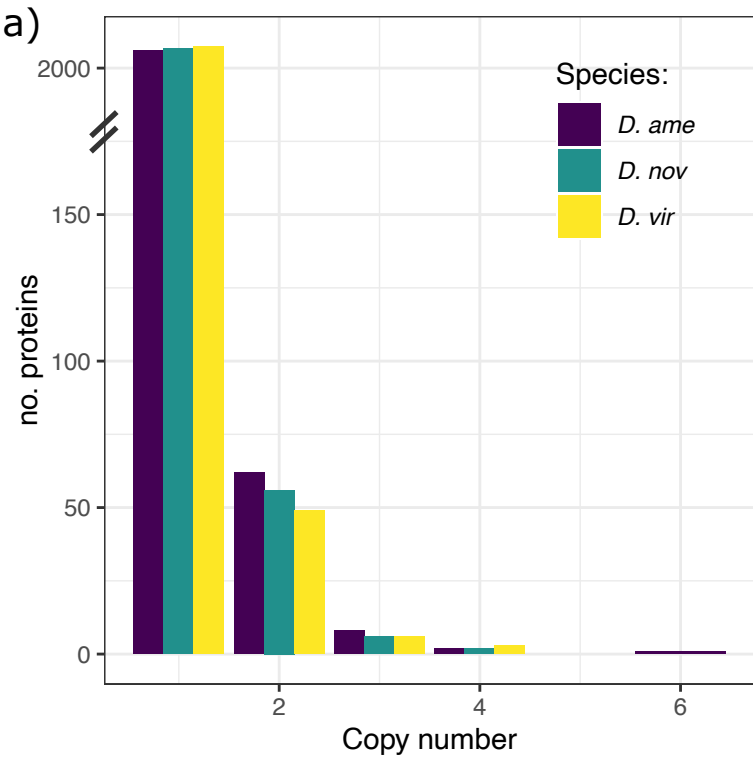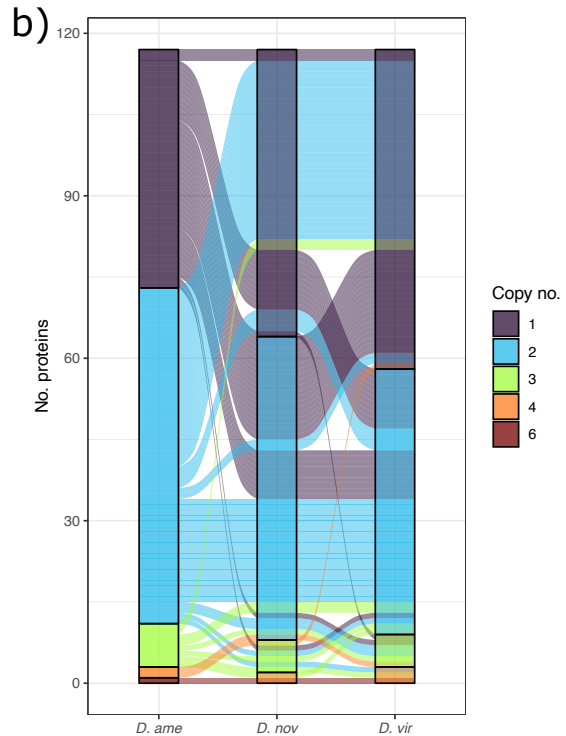

Supplement: Figure S2 — Representation of proteins identified in the current study belonging to orthogroups.A, number of proteins belonging to single copy or multi-copy gene families. Note the axis break on the y-axis to improve clarity of the number of multi-copy genes. B, relative copy number position between species for proteins belonging to a gene family in one or more species (n = 117). Lines show copy number relationship for each protein between species. [file mmc5.pdf]

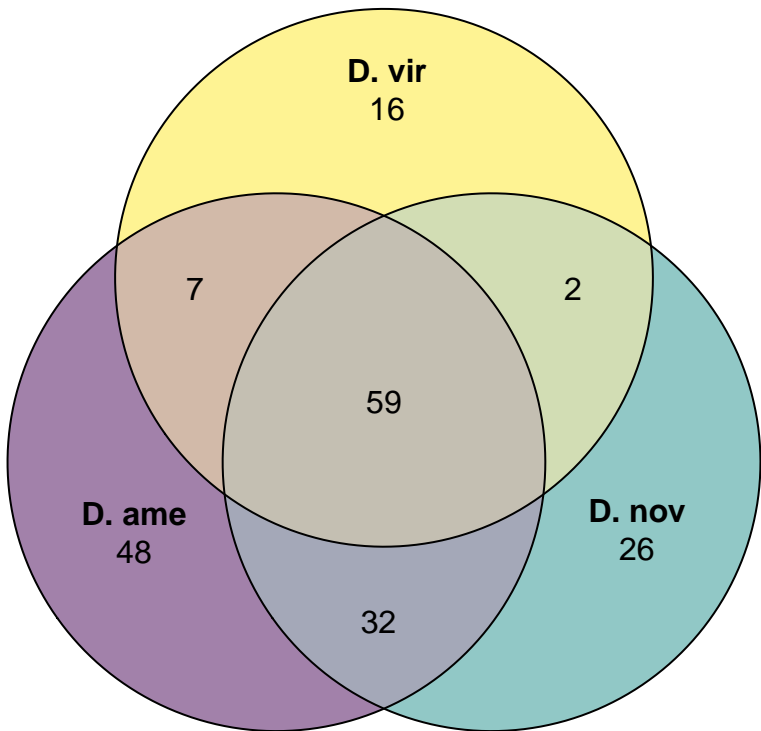

Supplement: Figure S3 — Shared and unique ejaculate proteins between species, including those without orthologs, using each species database. [file mmc6.pdf]

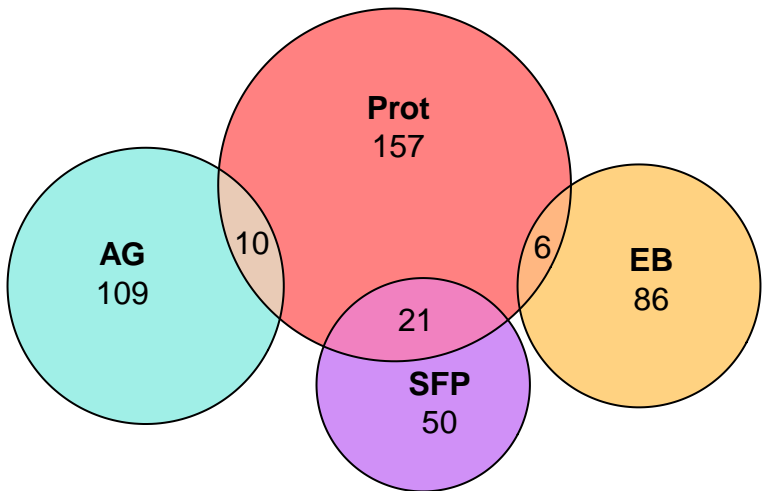

Supplement: Figure S4 — Overlap between proteins identified in the current study (Prot) with accessory gland (AG), ejaculatory bulb (EB), or SFPs identified by Ahmed-Braimah et al. (36). [file mmc7.pdf]

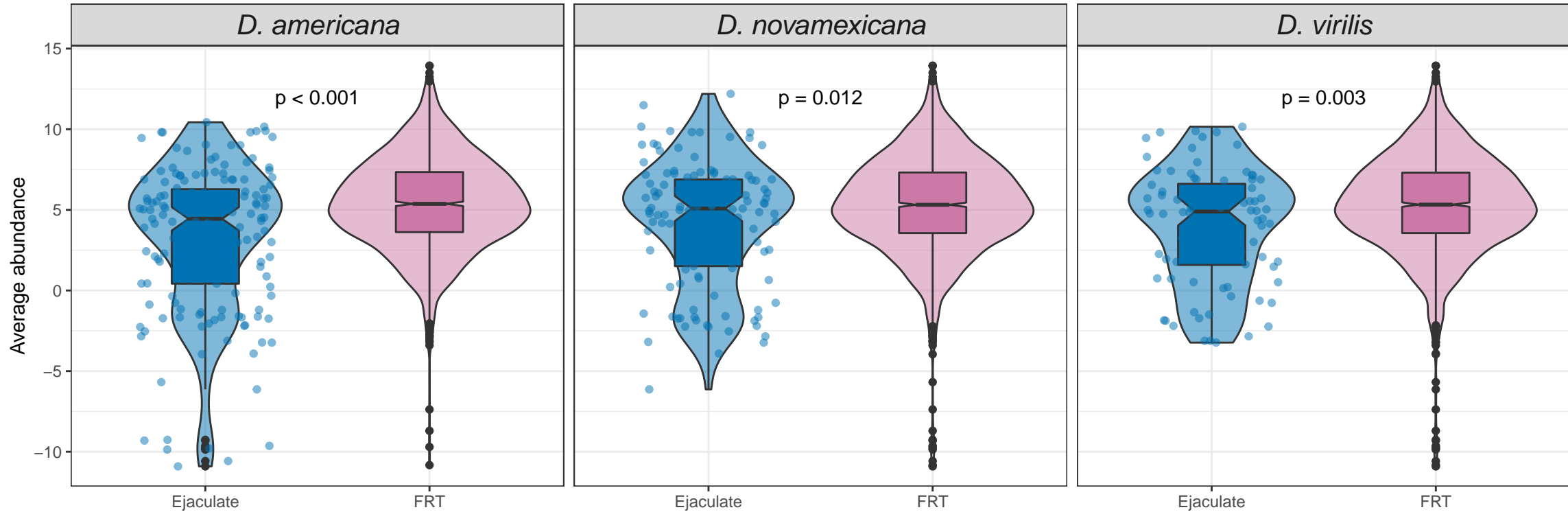

Supplement: Figure S5 — Abundance of ejaculate proteins vs. remaining female reproductive tract (FRT) proteins in each species. Points are individual proteins. P-values obtained from Mann-Whitney U tests. [file mmc8.pdf]

normalised abundance

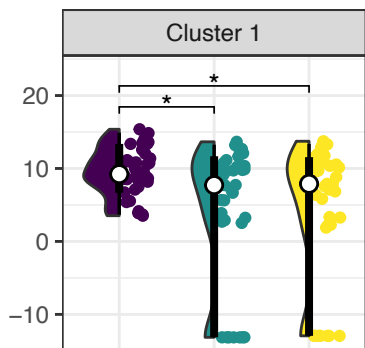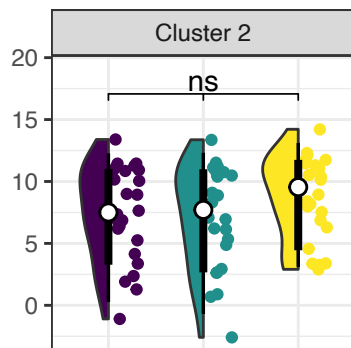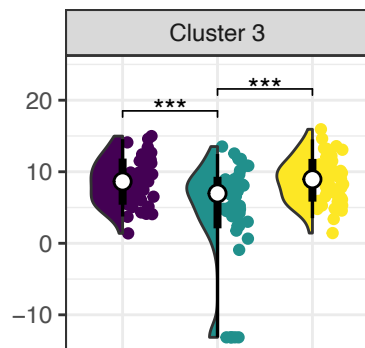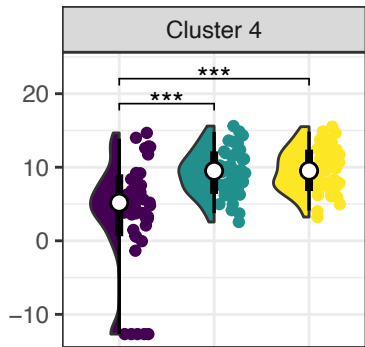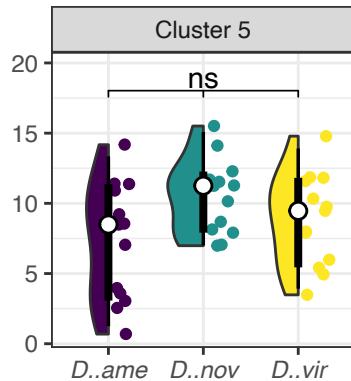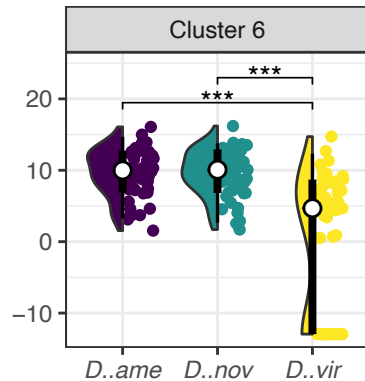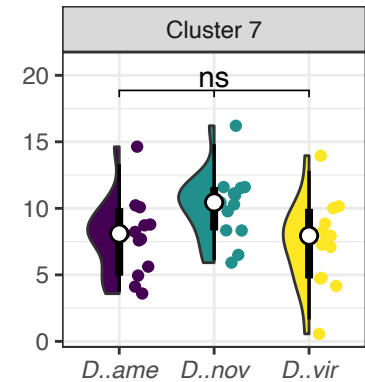

Supplement: Figure S6 — Abundance of ejaculate proteins in each k-means cluster (k = 7), 5 of which distinguished groups of proteins differing in abundance between species. Shown are normalised abundances for ejaculate proteins in each cluster. Large white points show the mean and small points are individual proteins. Thick and thin black bars showing the 66% and 95% confidence intervals, respectively. P-values indicate results from post-hoc Tukey's honest significant difference tests after analysis of variance performed on each cluster; ns: non-significant; ∗p < 0.05; ∗∗p < 0.01; ∗∗∗p < 0.001. [file mmc9.pdf]

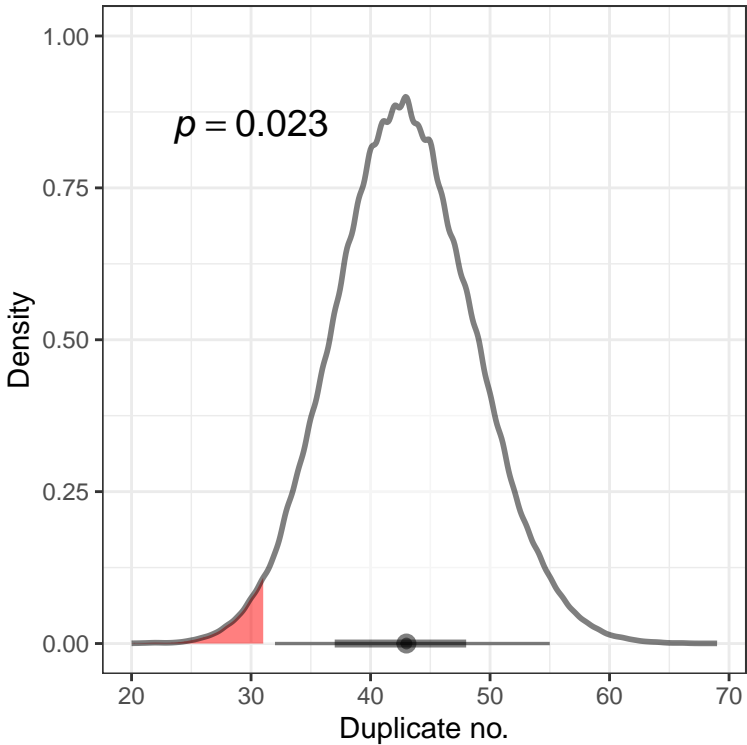

Supplement: Figure S7 — Density plot for permutation test sampling random draws without replacement of single copy or duplicate proteins from the entire proteome.P-value calculated as the total number of simulated draws (no. draws = 99,999) greater than or equal to the observed number of proteins belonging to multi-copy gene families (n = 31). [file mmc10.pdf]

normalised abundance

Cluster 1

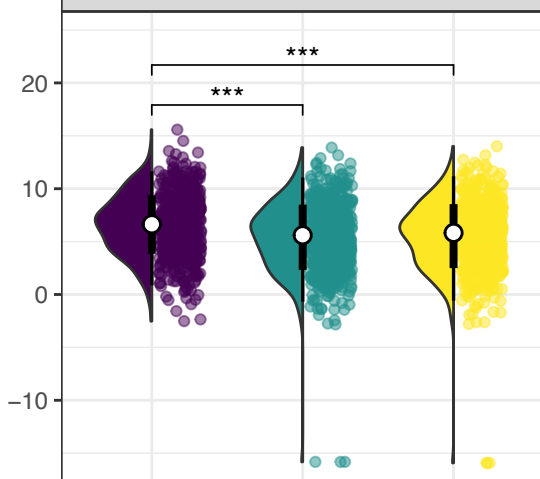

Cluster 2

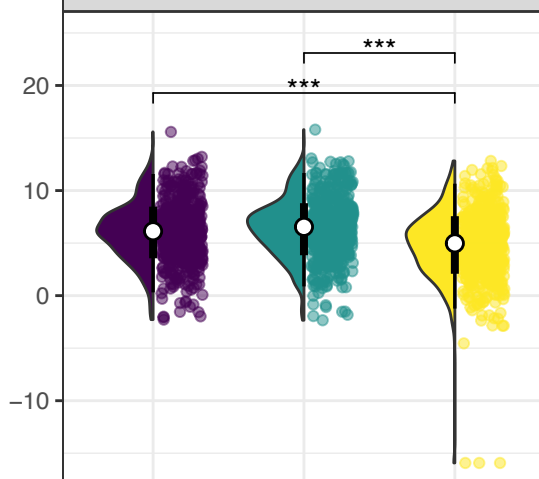

Cluster 3

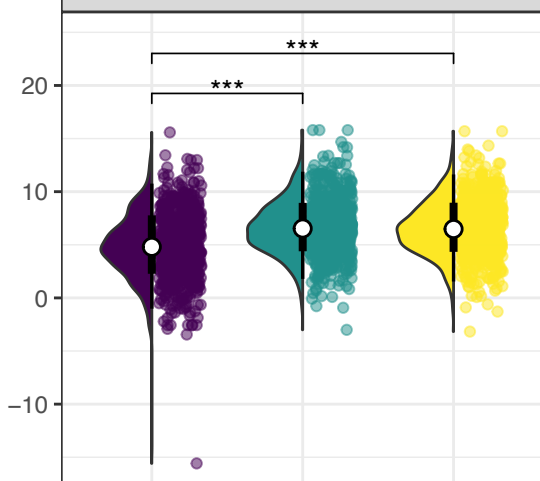

Cluster 4

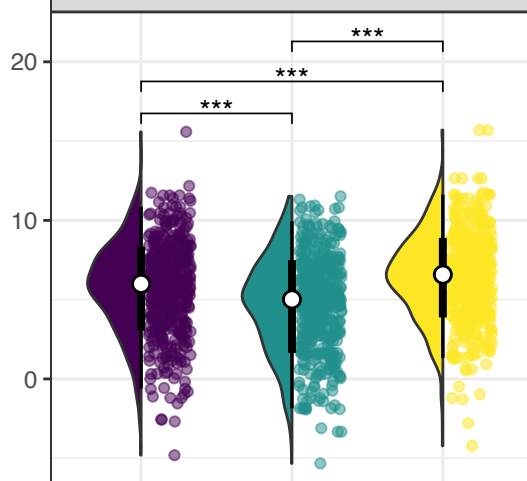

Species

*D. ame*

*D. nov*

*D. vir*

*D. ame*

*D. nov*

*D. vir*

Supplement: Figure S8 — Abundance of female reproductive tract proteins in each cluster. K-means clustering was used to separate proteins into clusters (k = 4), which distinguished groups of proteins differing in abundance between species. White points show the mean with thick and thin black bars showing the 66% and 95% confidence intervals, respectively. p-values represent results from post-hoc Tukey's honest significant difference tests after analysis of variance performed on each cluster; ns: non-significant; ∗p < 0.05; ∗∗p < 0.01; ∗∗∗p < 0.001. [file mmc11.pdf]

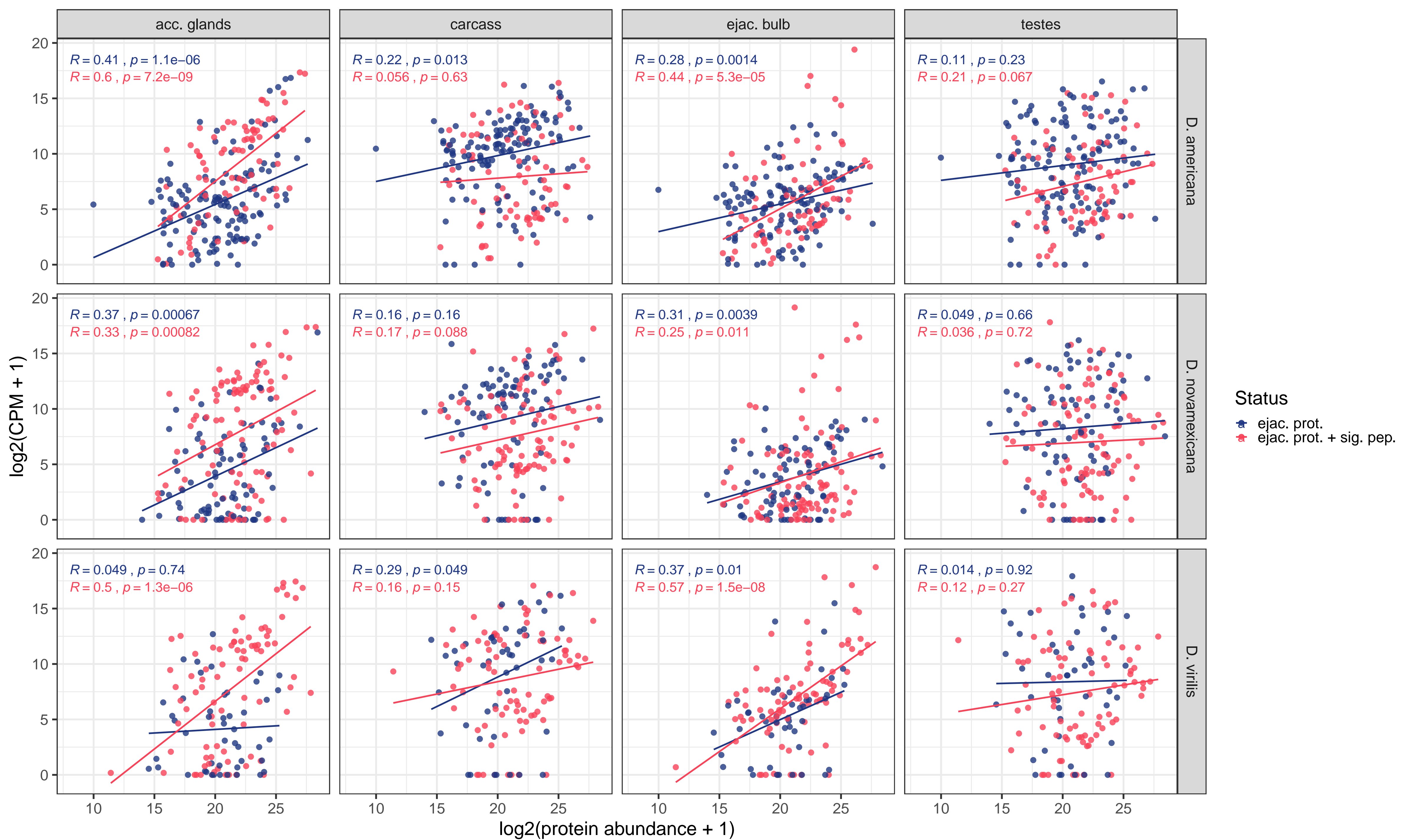

Supplement: Figure S9 — Correlation between mRNA abundance and protein abundance for ejaculate proteins across the three species and four male tissues: accessory glands, ejaculatory bulb, testes, and gonadectomized carcass. Correlation coefficients and p-values are indicated within each panel and separately for ejaculate proteins and ejaculate proteins with a predicted signal peptide. [file mmc12.pdf]
