# Supplementary material for: Evolutionary Quantitative Proteomics of Reproductive Protein Divergence in Drosophila
Source: Mol Cell Proteomics. 2023 Jun 28;22(8):100610. doi: 10.1016/j.mcpro.2023.100610 (PMC10407754; doi:10.1016/j.mcpro.2023.100610)
Supplement: Appendix [file mmc14.docx]

*APPENDIX - Database comparisons*

The rapid molecular evolution of reproductive proteins (99) poses a significant challenge for protein identification and quantitation with high throughput proteomics using liquid-chromatography tandem mass-spectrometry (LC-MS/MS) and data dependent acquisition (DDA). Most proteomics studies use a single query database to assign mass spectra to peptides and proteins, either using the proteome of one of the species of interest (34), the proteome of the closest available relative (33), or performing *in silico* prediction to generate a reference proteome (100). Using a single species query database for cross-species comparisons may suffer from problems akin to read mapping bias in short read RNA sequencing (RNA-seq) analysis pipelines (101). For instance, when using a protein database from another species, if a protein has undergone significant amino acid substitutions, spectra may be matched to an incorrect peptide or be unassigned completely, resulting in misidentification or inaccurate protein abundance estimation (102). Studies have revealed deep coverage of the sperm proteome across distantly related taxa after relatively long evolutionary time periods, likely aided by the majority of proteins in the sperm proteome being subject to evolutionary constraint (77,102–105). However, cross-species protein identification and quantitation using a single species query database may be more challenging for proteins that evolve more quickly on average, such as SFPs. Therefore, to assess the utility of different species query databases for protein identification and quantitation we performed analyses using each species proteome separately and compared the results. We predict greater precision using species-specific sequence information.

To assess variation in protein abundance between biological replicates using each species database we calculated the coefficient of variation (CV; (${sd}/\bar{\mu}$)) for each treatment (mated or virgin within each species). Mean CVs were generally lower for each treatment when using the corresponding species database, indicating more precise protein quantitation when using the corresponding species database (Fig. A1). To assess variation attributable to differences in quantitation between databases we then calculated CVs for each replicate across all three species databases. Mean CVs were higher than compared to within each database, indicating more variability in quantitation between each species database for the same replicate than between biological replicates using a single database (Fig. A2).

To complement this analysis, we compared the correlations in protein abundances between biological replicates of each treatment. If alternate species query databases are suitable for estimating protein abundances, then correlations between the same replicate using alternate species query databases should theoretically approach unity. Conversely, if mass-spectra are not assigned accurately when using an alternative species database, then abundance estimates will be less correlated between databases. Mean Pearson's correlations for each treatment were all > 0.98 when using a single species query database, indicating biological replicates behaved consistently (Fig. A3a,b). However, correlations were lower (range 0.73-0.80) when comparing protein abundances using alternate species databases to those obtained using species-specific query databases (Fig. A3a,b), suggesting alternate species databases provide less accurate protein abundance estimates.

We then tested for differences in abundance when using alternative species databases which revealed between 16-33% of proteins differed in estimated abundance. Proteins detected at significantly different abundances had elevated dN/dS (Mann-Whitney U test, all *p* < 0.019; Fig. A3c), as expected if amino acid substitutions reduce accuracy in peptide and protein assignment. Furthermore, proteins without reciprocal orthologs were found at lower average abundance using the *D. americana* (*p* = 0.003) and *D. novamexicana* (*p* = 0.024) databases (Fig. A3d), indicating less abundant proteins may be missed using an alternative species database.

We identified similar proportions of proteins that are more abundant in mated vs. virgin samples using all databases (Table 1). However, the combined database detected considerably more differentially abundant proteins between species for both ejaculate proteins and female reproductive tract proteins, while each species database identified similar proportions of differentially abundant proteins (Fig. A3e).

These analyses reveal the extent of bias that can be introduced when using divergent protein databases (i.e., inter-species databases) when querying mass spectra, and suggests that a species-specific query database is the best option for accurate protein identification and quantitation. Differences between species in the background proteome could also impact our results. However, such differences should be ameliorated by the close evolutionary relationship between species and tissue homology between samples analysed.

Figure A1. Coefficients of variation (CV) calculated for each treatment (mated vs. virgin for each species) using each species database.

Figure A2. Coefficients of variation (CV) calculated for each replicate across databases.

Figure A3. Database comparisons. a) Heatmap of average abundances for each treatment (mated and virgin) for each species using each species query database (n = 2642). b) Pearson's correlations (means ± standard error) comparing protein abundances quantified using each species query database. Correlations were calculated between replicates within each treatment for each species for comparisons within species, whereas correlations were calculated only between the same replicate when using alternate species query database. c) Mean dN/dS (± s.e.) comparing proteins showing differential abundance (SD; blue) between databases or not (NS; orange). d) Abundance of proteins identified using each database with- or without- reciprocal 1:1:1 orthologs; ns: non-significant; *: *p* < 0.05; **: *p* < 0.01. e) Proportion of proteins identified as ejaculate proteins (left); differentially abundant ejaculate proteins between species (middle); and female reproductive tract proteins between species (right). Proportions were calculated using the total number of differentially abundant proteins identified in each class divided by the total number of proteins used in each analysis (see Table 1). Note difference in Y-axis scales in (e). Abbreviations: First letter: A, *D. americana*; N, *D. novamexicana*; V, *D. virilis*. Second letter: M, mated; V, virgin.
